# Evaluation of image quality with four positron emitters and three preclinical PET/CT systems

**DOI:** 10.1186/s13550-020-00724-z

**Published:** 2020-12-10

**Authors:** Jarmo Teuho, Leon Riehakainen, Aake Honkaniemi, Olli Moisio, Chunlei Han, Marko Tirri, Shihao Liu, Tove J. Grönroos, Jie Liu, Lin Wan, Xiao Liang, Yiqing Ling, Yuexuan Hua, Anne Roivainen, Juhani Knuuti, Qingguo Xie, Mika Teräs, Nicola D’Ascenzo, Riku Klén

**Affiliations:** 1 Turku PET Centre, University of Turku, Turku, Finland; 2 Turku PET Centre, Turku University Hospital, Turku, Finland; 3 Department of Biomedicine, University of Turku, Turku, Finland; 4 RaySolution Digital Medical Imaging Co., Ltd, Ezhou, People’s Republic of China; 5 MediCity Research Laboratory, University of Turku, Turku, Finland; 6 School of Life Science and Technology, Huazhong University of Science and Technology, Wuhan, People’s Republic of China; 7 School of Software Engineering, Huazhong University of Science and Technology, Wuhan, People’s Republic of China; 8 Turku Center for Disease Modeling, University of Turku, Turku, Finland; 9 Department of Medical Physics and Engineering, Istituto Neurologico Mediterraneo NEUROMED I.R.C.C.S., Pozzilli, Italy; 10 Wuhan National Laboratory for Optoelectronics, Huazhong University of Science and Technology, Wuhan, China; 11 Department of Medical Physics, Turku University Hospital, Turku, Finland

**Keywords:** Preclinical imaging, NEMA, Image quality, PET/CT, Positron emission tomography, Small-animal

## Abstract

**Background:**

We investigated the image quality of ^11^C, ^68^Ga, ^18^F and ^89^Zr, which have different positron fractions, physical half-lifes and positron ranges. Three small animal positron emission tomography/computed tomography (PET/CT) systems were used in the evaluation, including the Siemens Inveon, RAYCAN X5 and Molecubes β-cube. The evaluation was performed on a single scanner level using the national electrical manufacturers association (NEMA) image quality phantom and analysis protocol. Acquisitions were performed with the standard NEMA protocol for ^18^F and using a radionuclide-specific acquisition time for ^11^C, ^68^Ga and ^89^Zr. Images were assessed using percent recovery coefficient (%RC), percentage standard deviation (%STD), image uniformity (%SD), spill-over ratio (SOR) and evaluation of image quantification.

**Results:**

^68^Ga had the lowest %RC (< 62%) across all systems. ^18^F had the highest maximum %RC (> 85%) and lowest %STD for the 5 mm rod across all systems. For ^11^C and ^89^Zr, the maximum %RC was close (> 76%) to the %RC with ^18^F. A larger SOR were measured in water with ^11^C and ^68^Ga compared to ^18^F on all systems. SOR in air reflected image reconstruction and data correction performance. Large variation in image quantification was observed, with maximal errors of 22.73% (^89^Zr, Inveon), 17.54% (^89^Zr, RAYCAN) and − 14.87% (^68^Ga, Molecubes).

**Conclusions:**

The systems performed most optimal in terms of NEMA image quality parameters when using ^18^F, where ^11^C and ^89^Zr performed slightly worse than ^18^F. The performance was least optimal when using ^68^Ga, due to large positron range. The large quantification differences prompt optimization not only by terms of image quality but also quantification. Further investigation should be performed to find an appropriate calibration and harmonization protocol and the evaluation should be conducted on a multi-scanner and multi-center level.

## Background

There is an increasing demand for standardization in preclinical positron emission tomography/computed tomography (PET/CT) studies, especially if performed in a multi-center or multi-system setting. Preclinical imaging studies have high requirements for image quality and quantification accuracy, which are dependent—beside other factors—on the accuracy of image reconstruction algorithms and data corrections [1, 2]. The available radiotracers with their unique physical properties, physics involved in the system hardware, data acquisition and reconstruction process and the physiology, among others, have a significant impact upon measurements performed in vivo [3]. Ensuring the reliability, reproducibility, validity and translatability of the preclinical data is of utmost importance [4]. Therefore, both the technical and non-technical factors that affect the quality and quantification of PET images should be carefully investigated.

While animal handling protocols have the greatest impact in preclinical studies, the characteristics of the used radiotracer also affect PET image quality [1]. Preferably, image quality should be studied with different systems in a standardized manner. For this purpose, phantom studies have been proposed to quantify the PET/CT system-specific differences in multi-system studies within an imaging center [2] and between several centers and systems [5]. A widely accepted PET protocol for system performance testing has been proposed by the National Electrical Manufacturers Association (NEMA), which includes a phantom and an image acquisition and analysis protocol for image quality evaluation.

The parameters such as image uniformity and recovery coefficients obtained using the NEMA NU4-2008 image quality protocol have been suggested to be used as metrics for performance standardization [2, 6, 7]. Recently, a multi-center study using a standard NEMA image quality phantom and 2-deoxy-2-[^18^F]fluoro-D-glucose ([^18^F]FDG) was performed, which showed that PET and PET/CT systems from a single vendor achieve comparable recovery coefficients, spill-over ratios and percentage standard deviations regarding performance values [4]. Therefore, it is possible to standardize the performance between single-vendor preclinical PET and PET/CT systems using the NEMA parameters and ^18^F.

As preclinical imaging is often performed by a wide variety of radiotracers, the image quality parameters should also be compared using multiple radionuclides with various physical properties. Radionuclides such as ^11^C, ^68^Ga, ^18^F and ^89^Zr are commonly used in small-animal PET imaging, with half-lives ranging from several minutes (20 min for ^11^C) to several days (3.27 days for ^89^Zr) [1]. Moreover, these radionuclides have different positron energies, positron fraction and range [8]. For example, positron ranges larger than intrinsic resolution might reduce both image quality and resolution of the PET images [9]. The difference in half-lives and positron fractions also affects the counting statistics, resulting to noise differences if a standard acquisition time is used. The presence of photons due to single emission could increase spill-over to regions of low activity, resulting in increased image noise [9]. Therefore, it is essential that image quality parameters are also evaluated using different radionuclides in a standardized fashion, to study both radionuclide- and system-dependent effects.

Previously, evaluations with different radionuclides on the Inveon and Focus 120 preclinical PET systems have been performed [9, 10]. Liu et al. studied the loss of resolution due to the different positron ranges of several radionuclides [10], while Disselhorst et al. performed an image quality evaluation of a single small-animal PET/CT with ^68^Ga, ^18^F, ^89^Zr and ^124^I [9]. Both reports emphasized that it is relevant to investigate preclinical PET/CT performances for different radionuclides, especially in regard to assessment of overall image quality [9, 10]. Further evaluations have been performed with various systems and radiotracers including ^18^F, ^68^Ga, ^64^Cu and ^11^C [11–14]. While previous studies have focused on evaluations with preclinical imaging systems of a single vendor, it would be of high interest to extend the investigation of the image quality parameters to a multi-radionuclide setting using different PET/CT systems. In this manner, the reproducibility of image quality parameters across radionuclides and systems on a baseline level using a similar acquisition and analysis protocol could be better investigated.

Our motivation was to extend these evaluations by performing assessment of image quality parameters with several radionuclides on three small animal PET/CT systems from different vendors, on a single scanner level. The evaluation was performed using four radionuclides, including ^11^C, ^68^Ga, ^18^F and ^89^Zr. The standard 20 min acquisition time specified by the NEMA protocol was used for ^18^F. Radionuclide-specific acquisition times to account for the physical half-life and positron fraction differences between the nuclei were used for ^11^C, ^68^Ga, and ^89^Zr. A NEMA image quality phantom was used and the preclinical NEMA NU 4-2008 performance protocol was followed, to determine the radionuclide-specific effects on the resulting image quality of each system separately. It is the first time, to our knowledge, that the effect of physical properties of different radionuclides on image quality parameters is investigated using several preclinical PET/CT imaging systems in a single-center setting.

## Methods

### Preclinical PET/CT systems

Three small animal PET/CT systems were evaluated, namely the RAYCAN Trans-PET/CT X5 (RAYCAN Technology, Suzhou, China), Inveon Multimodality PET/CT (Siemens Medical Solutions, Knoxville, TN, USA) and Molecubes β-cube (PET) and X-cube (CT) (MOLECUBES NV, Ghent, Belgium). The acquisition software versions at the time of the study were 1.0.1371, 2.0 and 1.5.2 for the RAYCAN, Inveon and Molecubes, respectively. All systems are located within one institute. The RAYCAN and Inveon systems are physically one system where the bed is moved automatically between PET and CT. The Molecubes system is based on two separates scanners where the bed needs to be physically transferred between PET and CT. Co-registration between PET and CT is performed using a rigid registration matrix, which is calculated as part of the system calibration.

The performance aspects of these systems are described in detail elsewhere [15–17]. The system peak sensitivities are 1.7%, 9.3% and 12.4%, and the reported resolutions of the systems are 1.9 mm, 1.8 mm and 1.1 mm for RAYCAN, Inveon and Molecubes, respectively [15–17]. A summary of the technical characteristics of the systems is provided in Table 1. We will refer these systems as RAYCAN, Inveon and Molecubes throughout the paper.

**Table 1:** Technical characteristics of the preclinical PET/CT systems involved in the evaluation [15–17]

System	Detector crystal	Detector size (mm)	Detector rings (N)	Ring diameter (cm)	Transaxial FOV (mm)	Axial FOV (mm)	Energy resolution (%)	Timing resolution (ns)	NEMA resolution (mm)	NEMA sensitivity (%)
RAYCAN	LYSO	1.89 × 1.89 × 13	26	19.2	130	53	15	5	1.9	1.7
Inveon	LSO	1.51 × 1.51 × 10	80	16.1	100	127	15	3.43	1.8	9.3
Molecubes	LYSO	25.4 × 25.4 × 8^*^	5	7.6	72	130	12	2.4	1.1	12.4

The resolution values are reported based on the filtered backprojection (FBP) reconstruction for all systems

^*^Monolithic detector

The calibration of the systems is performed regularly according to the protocol provided by the vendor. The calibration protocols use ^18^F and no radionuclide-specific calibration is performed. Specifically, the calibration procedure for Inveon was performed using a 50 mL syringe and using 20 MBq of ^18^F. For Molecubes, 3 mL, 10 mL and 20 mL syringes and 5 MBq of ^18^F are used. For RAYCAN, a 4 cm diameter cylindrical phantom with 13 MBq of ^18^F is used. All of the calibrations of the systems are performed using a single Veenstra VDC-405 dose calibrator (Veenstra Instruments, Joure, The Netherlands). All recorded doses were also measured using this dose calibrator.

The default energy windows and coincidence timing windows were used on each system. The energy windows were 350–650 keV on the RAYCAN, 350–650 keV on the Inveon and 358–664 keV on the Molecubes system. The timing windows were 5 ns, 3.44 ns and 10 ns, respectively.

### Radionuclides used for PET imaging

The radionuclides used were [^11^C]acetate, [^68^Ga]chloride, [^18^F]FDG, and [^89^Zr]oxalate. The physical characteristics of the selected radionuclides are summarized in Table 2. The radionuclides have physical half-lives ranging from 20.4 min to 3.27 d, while the maximum positron range in water varies from 2.4 to 9.2 mm.

**Table 2:** Physical properties of different radionuclides used for PET image quality evaluation [8]

Radionuclide	Physical half-life	Branching ratio	Max β^+^ energy (MeV)	Maximum β^+^ range in water (mm)	Prompt gamma branching ratio and energy
^11^C	20.4 min	0.998	0.960	4.2	–
^68^Ga	67.8 min	0.877	1.899	9.2	0.03, 1.08 meV
^18^F	109.8 min	0.969	0.634	2.4	–
^89^Zr	3.27 d	0.227	0.902	3.8	0.99, 909.2 keV

### NEMA image quality phantom

A standard NEMA image quality phantom was used [6]. The phantom was shipped originally with the RAYCAN system and was manufactured by RAYCAN (RAYCAN Technology, Suzhou, China), according to the NEMA specifications. The phantom has a length of 50 mm and diameter of 30 mm, where the main compartment is made in one part of solid plastic. The phantom consists of three regions, which can be used to analyze different aspects of image quality. A construction scheme with a photograph of the phantom can be found from Additional file 1: Fig. S1.

The first 20 mm of the phantom consists of 5 rods with diameters of 1, 2, 3, 4, and 5 mm, which are embedded in plastic to form a cold background. During phantom preparation, the rods are simply filled with water connected with the main cavity and contain the same concentration of radioactivity with each other and the main cavity. The rods are used to determine the recovery coefficient (Eq. 1). The central region of the phantom consists of a large uniform compartment, used to determine image uniformity and changes in the activity distribution due to noise and other effects (Eqs. 2 and 3).

The phantom also contains two cylinders with 8 mm of inner diameter and 14 mm in length. One of the cylinders is filled with air while the other is filled with water without radioactivity added. Neither of these cylinders is connected with the radioactivity in the main phantom volume, therefore they are representing two cold volumes on hot background. The cylinders are used to define the spill-over ratios in air and water (Eq. 4).

### Image acquisition protocol

The standard NEMA protocol is designed specifically for ^18^F, where a 20 min emission scan duration with an initial activity of 3.70 MBq (± 5% accuracy) is recommended [6]. To record a similar amount of total coincidence events than for ^18^F, either the acquisition duration or the initial activity needs to be adjusted to different positron fractions and physical half-lives of the other radionuclides [9, 12, 13]. In this manner, the difference in counting statistics between each radionuclide can be minimized. We adjusted the total scan duration for all of the non-^18^F radionuclides. We chose to increase the acquisition time instead of activity, as increased activity might result in changes in scatter and randoms rates with increased system dead-time. These effects could introduce variations in the results, although they are expected to be low with this level of activity.

For determining radionuclide-specific acquisition times, we determined the acquisition time first on one system (Inveon) and fixed the duration on other systems (RAYCAN, Molecubes). Whereas this protocol does not account for the sensitivity differences between the systems, it allows to achieve a comparative evaluation of image quality parameters with different radionuclides within an individual PET/CT system.

Only on the Inveon system, it was possible to acquire the emission scan with a predefined number of counts. We set the system to acquire 190 M total events (prompts + delays), which corresponded to 20 min of emission duration for ^18^F. For other radionuclides, the Inveon system was set to acquire 190 M total events (prompts + delays) and the final acquisition time was noted. Thereafter, the same acquisition duration as determined on the Inveon system was then used for the RAYCAN and Molecubes systems, as these systems did not have an option to acquire by a set number counts. For each system, we extracted the number of total events corresponding to prompts + delays to confirm the amount of collected events between each radionuclide. The total number of counts collected with each system and the acquisition duration for each radionuclide can be found from Table 3. The experimental acquisition times were close to the theoretical acquisition times calculated in the paper of Disselhorst et al. [9].

**Table 3:** Number of events recorded on each PET/CT system and the final acquisition times used in the evaluation of different nuclei

PET/CT system	The number of events (prompts + delays) for each isotope			
	[^18^F]FDG	[^11^C]acetate	[^68^Ga]chloride	[^89^Zr]oxalate
RAYCAN	37.96 M	41.07 M	38.37 M	39.59 M
Inveon	185.30 M	190.60 M	192.32 M	178.04 M
Molecubes	382.43 M	407.83 M	394.38 M	480.08 M
Acquisition times	20 min	31 min 41 s	25 min 32 s	72 min

For image acquisition, the phantom was positioned on the scanner bed, oriented in the axial direction and centered in the field-of-view (FOV), using built-in lasers for guidance and CT scout images where available. The phantom was centered on the homogenous compartment with careful positioning to the phantom midline, which was marked to ensure repeatable positioning of the phantom between measurements. On all systems, a CT scan of the phantom was acquired for localization and attenuation correction for PET. The CT scan was acquired using the default parameters on each system which are as follows: 50 kVp and 1 mA for RAYCAN, 80 kVp and 0.5 mA for Inveon and 50 kVp and 0.1 mA for Molecubes, respectively. Thereafter, a PET scan over the entire phantom was performed with the radionuclide-specific acquisition time.

The radiotracer doses and activity concentrations at scan start times are shown in Table 4. For ^11^C, ^18^F and ^68^Ga acquisitions, the phantom was filled multiple times and was left to decay before proceeding to subsequent measurements. Before each measurement, the phantom was checked for and cleaned of any activity remaining from previous use. The total volume of the phantom regions filled with activity was measured as 22 mL according to phantom weight.

**Table 4:** Doses and activity concentrations at scan start times with acquisition times of the radiotracers on different PET/CT systems

PET/CT system	Dose (MBq)/activity concentration (kBq/mL) at scan start time for each radionuclide			
	[^11^C]acetate	[^68^Ga]chloride	[^18^F]FDG	[^89^Zr]oxalate
RAYCAN	3.69/167.73	3.72/169.55	3.70/168.18	3.68/167.27
Inveon	3.40*/154.55	3.73/169.55	3.76/170.91	3.68/167.27
Molecubes	3.69/167.73	3.72/169.09	3.70/168.18	3.68/167.27

*Activity outside the specified limits of NEMA (3.70 MBq ± 5%)

### PET image reconstruction

All PET images were reconstructed using three-dimensional (3D) iterative reconstruction algorithms, using the default settings for histogramming and reconstruction. All available data corrections were applied, including dead-time, decay, normalization, geometric effects attenuation. Molecubes and Inveon apply the single scatter simulation method for scatter correction [18] and a delayed window method for randoms correction [19]. RAYCAN does not apply scatter or randoms correction. For attenuation correction, all the three scanners implement CT-based attenuation correction.

The reconstruction algorithms were 3D ordered-subset expectation maximization (3D-OSEM) with point spread function correction for RAYCAN [20], shifted Poisson model maximum a posteriori (SP-MAP) for Inveon [21] and a graphics processing unit (GPU)-based 3D-OSEM reconstruction for Molecubes [16]. No point spread function correction (3D-OSEM-PSF) was available on Inveon or Molecubes, whereas on the RAYCAN 3D-OSEM with point spread function correction was the only iterative algorithm available. The reconstruction parameters used and data corrections implemented for each system are summarized in Table 5.

**Table 5:** Image reconstruction parameters and data corrections implemented on the PET/CT systems

System	Reconstruction algorithm	Matrix size	Pixel size	Iterations/ Subsets	Post-filter	β-value/prior penalty	Data corrections	PSF applied
RAYCAN	3D-OSEM-PSF	280 × 280 × 100	0.5 × 0.5 × 0.5	2/12	Low	–	AC	Yes
Inveon	SP-MAP	128 × 128 × 159	0.8 × 0.8 × 0.8	18 /16	–	0.00427838	AC, SC, RC	–
Molecubes	3D-OSEM	192 × 192 × 384	0.4 × 0.4 × 0.4	30/7	–	–	AC, SC, RC	–

*AC* attenuation correction, *SC* Scatter correction, *RC* randoms correction

### Evaluation of image quality using the NEMA image quality phantom

The NEMA image quality phantom data was analyzed using the protocol specified in the NEMA NU 4-2008 standard [6]. The protocol involved evaluation of the recovery coefficient, image uniformity and spill-over effects by using in-house developed software in MATLAB2015b. These parameters have been proposed to be used as a metric for harmonization [1, 2]. A short description of the image quality metrics is given below.

The recovery coefficient determines the ability of an imaging system to recover contrast in small targets and reflects resolution. The recovery coefficient is theoretically limited to a value between 0 and 1, with values closer to 1 representing higher activity recovery, while values over 1 are considered as overestimation. The image uniformity and the percentage standard deviation in the uniform region are measures of image noise or other effects affecting the homogeneity of tracer distribution in that region. The smaller the value for percentage standard deviation, the smaller is the variation in the image, representing reduced noise. The spill-over ratio in both water and air represents the remaining contribution of scatter, positron range, randoms and other physical effects in the cold regions, as some activity will be spilled over in these regions The spill-over ratio is theoretically limited to values of 0 to 1, where values close to 0 indicate the smallest amount of spill-over.

To determine the recovery coefficient, the image slices over the central 10 mm length of the phantom rods were averaged to obtain one average slice of the rods. Circular regions of interest (ROIs) were drawn around each rod with diameters of twice the size of the physical diameter of the rods. From the ROIs, the maximum values were measured and the location of the maximum pixel coordinates was determined. The pixel coordinates were then used to create line profiles along the rods in the axial direction. To calculate the percent recovery coefficient (%RC), the pixel values in each line profile were divided by the mean activity measured from the uniform region to determine the mean %RC for each rod as:

1 $$\% {\text{RC}} = {\text{Mean}}_{{{\text{line}}\,{\text{profile}}}} /{\text{Mean}}_{{{\text{uniform}}{.}}} \times 100 \% ,$$

where ${\text{Mean}}_{{{\text{line}}\,{\text{profile}}}}$ corresponds to the mean activity of the line profile and ${\text{Mean}}_{{{\text{uniform}}{.}}}$ corresponds to the mean activity in the uniform region. Thereafter, the percent standard deviation of the recovery coefficients $(\% {\text{STD}}_{{{\text{RC}}}} )$ for each rod was determined from Eq. 2:

2 $$\% {\text{STD}}_{{{\text{RC}}}} = 100 \times \sqrt {({\text{STD}}_{{{\text{line}}\,{\text{profile}}}} /{\text{Mean}}_{{{\text{line}}\,{\text{profile}}}} )^{2} + ({\text{STD}}_{{{\text{uniform}}}} /{\text{Mean}}_{{{\text{uniform}}}} )^{2} } ,$$

where the mean and standard deviation were calculated from individual line profiles (${\text{Mean}}_{{{\text{line}}\,{\text{profile}}}}$ and ${\text{STD}}_{{{\text{line}}\,{\text{profile}}}}$) and the uninform region of the phantom (${\text{Mean}}_{{{\text{uniform}}}}$ and ${\text{STD}}_{{{\text{uniform}}}}$).

Uniformity was measured by drawing a 22.5 mm diameter and 10 mm long cylindrical volume of interest (VOI) over the center of the uniform region. The mean and percentage standard deviation (%*SD*) of the activity concentration were measured.

In addition, while the NEMA standard does not specify a measurement of the absolute quantification accuracy in the phantom, we calculated the percentage difference to the calculated activity concentration at scan start time in relation to activity concentration measured from the phantom uniform compartment. The percentage difference $\% \Delta$ was calculated as:

3 $$\% \Delta = \frac{{{\text{Mean}}_{{{\text{uniform}}{.}}} - A_{0} }}{{A_{0} }} \times 100,$$

where $A_{0}$ corresponds to the calculated activity concentration in kBq/mL at scan start time.

The spill-over of activity in the water and air-filled cylindrical inserts was defined by drawing VOIs of 4 mm in diameter and 7.5 mm length over the cylindrical inserts. The spill-over ratio (*SOR*) was calculated as the ratio of the mean of each cold region to the mean of the uniform region, defined as:

4 $${\text{SOR}} = {\text{Mean}}_{{{\text{cold}}}} /{\text{Mean}}_{{{\text{uniform}}{.}}}$$

The percent standard deviation $(\% {\text{STD}}_{{{\text{SOR}}}} )$ in the water- and air-filled rods was calculated in the same manner as the $\% {\text{STD}}$ of the recovery in Eq. 2, using the standard deviation and the mean calculated from the cold regions versus the uniform region.

## Results

**Fig. 1:**
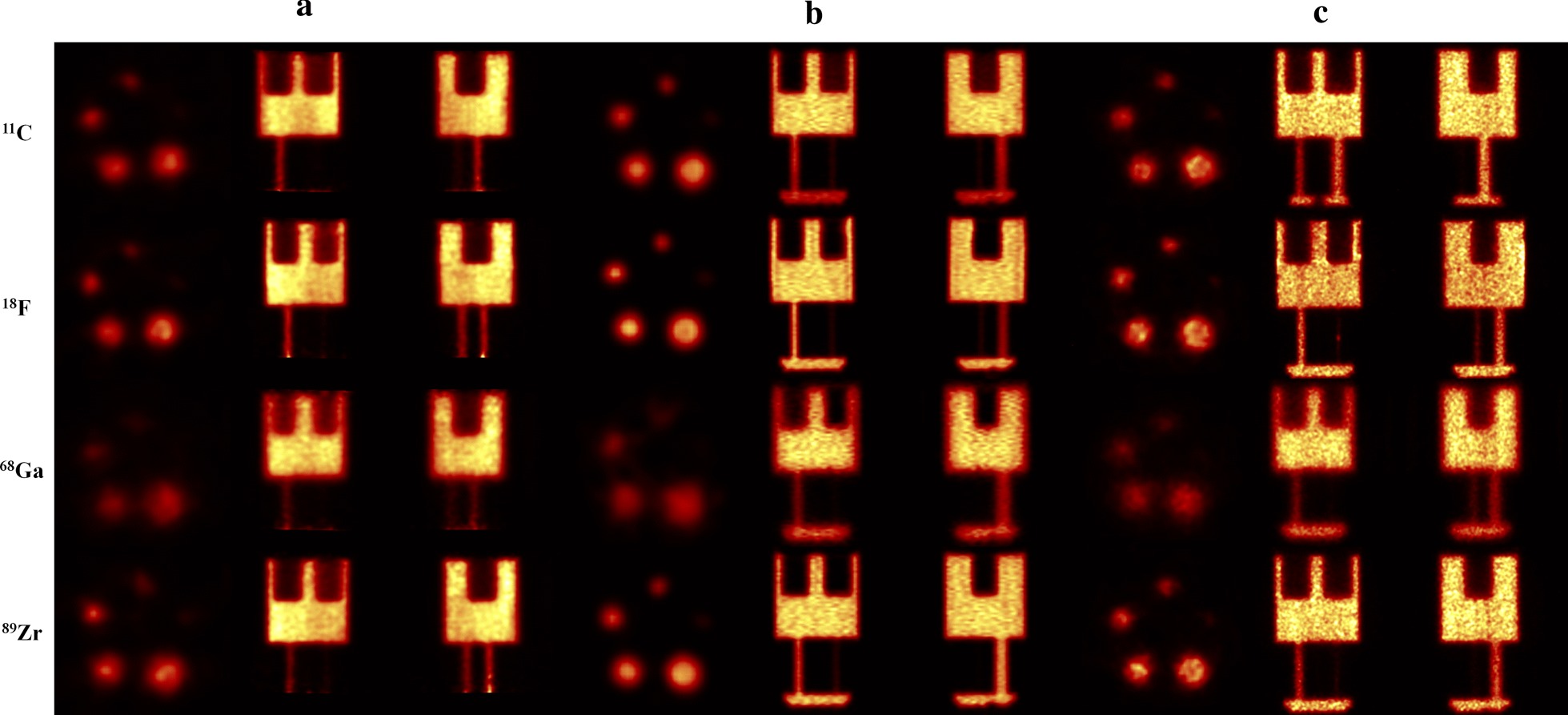
Image quality from one plane in the rod region of the NEMA phantom with different preclinical PET/CT systems. The systems shown are **a** RAYCAN, **b** Inveon and **c** Molecubes. Slight differences in the noise level between various radionuclides in the images within a system can be seen. The ^18^F and ^89^Zr have similar whereas appearance, whereas the positron range is the major contributing factor to the blurred appearance of the images with ^68^Ga. All images have been scaled between 0 and 1.25 times the mean activity of the uniform compartment

Figure 1 shows the transverse, sagittal and coronal views of the phantom with different radionuclides and systems. It can be seen that ^11^C, ^18^F and ^89^Zr have similar image quality across different imaging systems, while ^68^Ga shows the poorer image quality, independent of the system. Especially the rod section of the phantom is blurred with ^68^Ga.

**Fig. 2:**
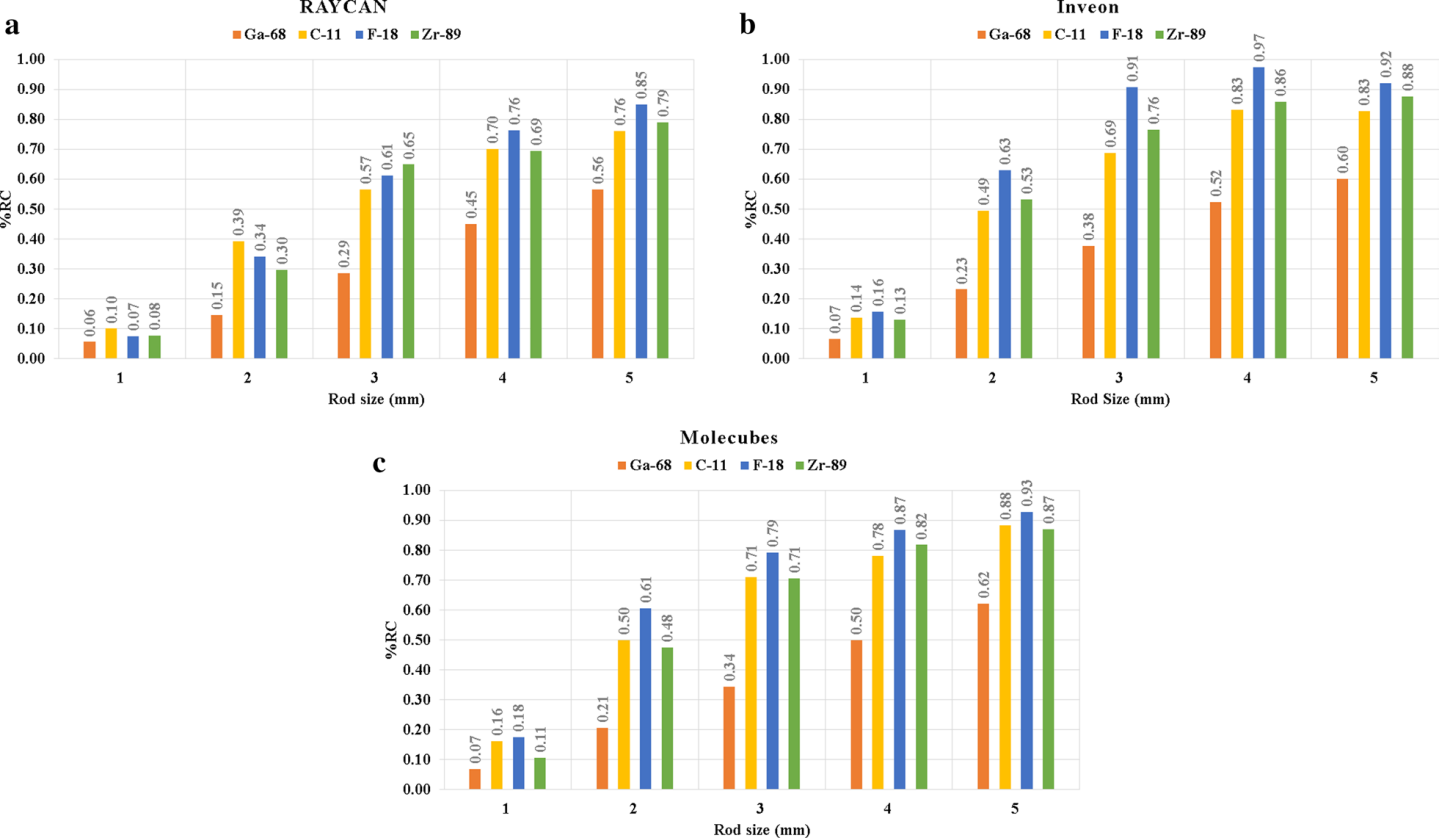
Recovery coefficients (%RC) with different radionuclides with the **a** RAYCAN, **b** Inveon and **c** Molecubes systems. The maximal recovery is achieved with ^18^F. ^11^C and ^89^Zr are close to the values with ^18^F. ^68^Ga shows the lowest recovery on all systems and rod sizes

The results from the %*RC* evaluation with all systems and radionuclides are shown in Fig. 2. Radionuclide-specific differences can be seen, which are also reflected across the systems to a degree. Maximum *RCs* with rod sizes from 1 to 5 mm were measured on the Inveon system using ^18^F (from 0.16 to 0.92), from rod sizes from 1 to 5 mm on the Molecubes system using ^18^F (from 0.18 to 0.93) and from rod sizes of 4 mm to 5 mm on the Raycan system using ^18^F (from 0.76 to 0.85). The lowest %*RC* values were measured with 1 mm rod sizes across all systems with ^68^Ga (range 0.06–0.07). The maximum %*RC* using ^68^Ga with 5 mm rod size was also the lowest of all radionuclides across all systems (range 0.56–0.62). In terms of %*RC* with rod sizes from 2 to 5 mm, ^11^C and ^89^Zr had *RCs* in between ^18^F and ^68^Ga nearly on all systems (Fig. 2). The ${\text{RC}}s$ were very similar between ^11^C and ^89^Zr across all rod sizes.

**Fig. 3:**
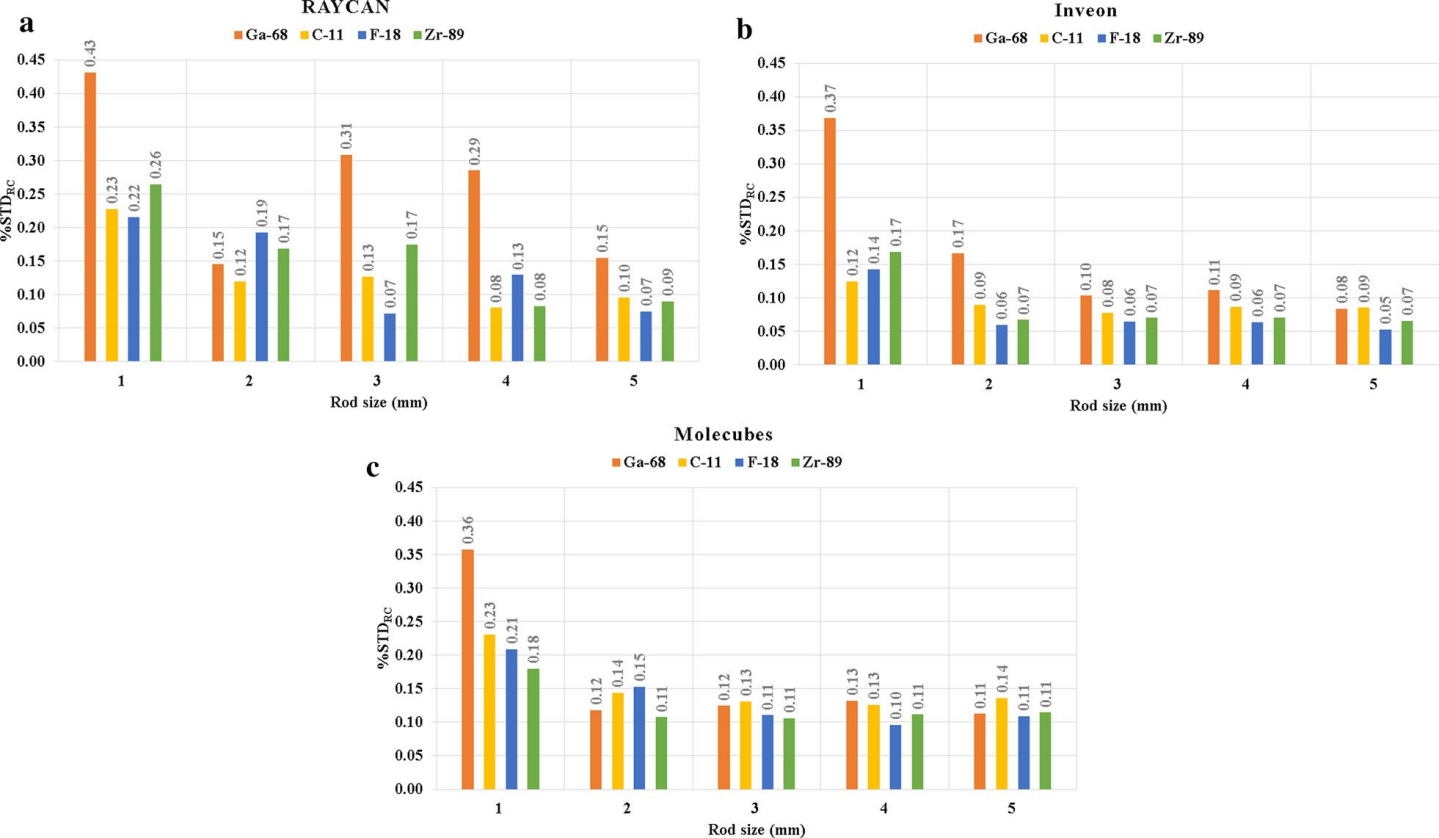
Percentage standard deviation $\left( {\% {\text{STD}}_{{{\text{RC}}}} } \right)$ with different rod sizes and radionuclides with the **a** RAYCAN, **b** Inveon and **c** Molecubes systems. Most variability in the $\% {\text{STD}}_{{{\text{RC}}}}$ is seen with ^68^Ga with rod size of 1 mm on the Molecubes system and with rod size of 1 mm and 2 mm on the Inveon system, reflecting system resolution. The RAYCAN system has more variability between radionuclides, where ^68^Ga shows the highest variability on nearly all rod sizes

The results from the $\% {\text{STD}}_{{{\text{RC}}}}$ evaluation with all systems and radionuclides are shown in Fig. 3. The largest variability was seen in the $\% {\text{STD}}_{{{\text{RC}}}}$ with ^68^Ga across all systems (0.08 to 0.43). Lowest $\% {\text{STD}}_{{{\text{RC}}}}$ were seen with ^18^F and ^89^Zr with the Inveon system (range 0.06–0.05 for ^18^F, 0.07 to 0.07 for ^89^Zr) from rod sizes of 2 to 5 mm and Molecubes from rod sizes of 3 to 5 mm (range 0.15–0.11 for ^18^F, 0.11 to 0.11 for ^89^Zr). Radionuclide-specific variation was highest with the RAYCAN system, with no clear trend between other radionuclides, with the exception of ^68^Ga producing highest $\% {\text{STD}}_{{{\text{RC}}}}$ nearly across all rod sizes.

Table 6 contains the results from the uniformity evaluation. The differences in %*SD* between the nuclides were 1.1%, 2.2% and 1.3% for RAYCAN, Inveon and Molecubes. ^18^F showed a low %*SD* with the Inveon (4.85%), Molecubes (7.39%) and RAYCAN system (6.03%). ^18^F and ^11^C had similar %*SD* for the RAYCAN system (approximately 6%). ^68^Ga, ^89^Zr and ^18^F had similar %*SD* for the Molecubes system (approximately 7%).

**Table 6:** The mean activity (kBq/mL) and the percentage standard deviation (%SD) measured from the uniform compartment of the phantom

PET/CT system	^11^C Mean activity (kBq/mL)	^11^C %SD	^68^Ga Mean activity (kBq/mL)	^68^Ga %SD	^18^F Mean activity (kBq/mL)	^18^F %SD	^89^Zr Mean activity (kBq/mL)	^89^Zr %SD
RAYCAN	190.13	5.81	168.90	6.91	182.57	6.03	196.61	6.24
Inveon	171.57	5.99	170.16	7.05	185.50	4.85	205.30	5.69
Molecubes	186.67	8.46	143.94	7.16	174.46	7.39	154.20	7.34

Table 7 contains the results from the evaluation of quantification accuracy, where the results varied significantly across nuclides and systems. Variation was large with ^89^Zr (range − 7.82 to 22.73%) and ^68^Ga (range − 14.87 to 0.36%), whereas ^11^C and ^18^F showed a positive bias with all systems (range 11.01–13.36% for ^11^C, range 3.73–8.56% for ^18^F). Surprisingly, ^68^Ga showed the best accuracy on the Inveon and the RAYCAN system with maximum errors of 0.36% and -0.38%. The best quantification accuracy with ^18^F was seen on the Molecubes system (3.73% error). The largest quantification errors were seen with ^89^Zr on the Inveon system (22.73%) and ^89^Zr on the RAYCAN system (17.54%) and ^68^Ga on the Molecubes system (-14.85%). Image quantification differences were smallest with ^18^F (range 3.73–8.56%) and ^11^C between the systems (range 11.01–13.36%).

**Table 7:** Percentage difference (%Δ) of the mean activity in the uniform compartment of the phantom compared to the reference activity measured at scan start time

PET/CT system	^11^C %Δ	^68^Ga %Δ	^18^F %Δ	^89^Zr %Δ
RAYCAN	13.36	− 0.38	8.56	17.54
Inveon	11.01	0.36	8.54	22.73
Molecubes	11.29	− 14.87	3.73	− 7.82

The results from the *SOR* and $\% {\text{STD}}_{{{\text{SOR}}}}$ evaluation with all systems and radionuclides can be found from Table 8. SOR showed more variation between the systems and radionuclides, especially in the water compartment. Both ^11^C and ^68^Ga showed the highest spill-over ratios in water (range 0.16–0.27 for ^11^C and 0.09 to 0.32 for ^68^Ga), while ^18^F and ^89^Zr had the lowest SOR in water (0.05 and < 0.01 on Inveon, 0.26 and 0.25 on RAYCAN, 0.07 and 0.07 on Molecubes). Highest SOR in air was measured with ^68^Ga on RAYCAN (0.25) and Inveon (0.06), respectively, and with ^11^C on Molecubes (0.13). The lowest SOR in air were measured on the Inveon system (range 0.01–0.06) and the highest were measured with the RAYCAN system (range 0.15–0.25). For the RAYCAN system, the data was not corrected.

**Table 8:** Spill-over ratios (SOR) with the percentage standard deviation ($\% {\text{STD}}_{{{\text{SOR}}}}$) in parenthesis measured from the phantom water and air compartment

SOR ($\% {\text{STD}}_{{{\text{SOR}}}}$)	^11^C	^68^Ga	^18^F	^89^Zr
RAYCAN*				
Water	0.27 (6.83)	0.32 (8.75)	0.26 (8.28)	0.25 (8.74)
Air	0.16 (8.54)	0.25 (10.42)	0.15 (8.97)	0.16 (7.64)
Inveon				
Water	0.16 (10.10)	0.09 (34.01)	0.05 (27.75)	< 0.01 (240.08)
Air	0.01 (46.04)	0.06 (55.52)	0.03 (29.03)	0.01 (106.38)
Molecubes				
Water	0.17 (19.50)	0.12 (13.78)	0.07 (15.85)	0.07 (16.71)
Air	0.13 (13.04)	0.09 (13.78)	0.07 (12.14)	0.06 (13.61)

*System has no data corrections for randoms and scatter

$\% {\text{STD}}_{{{\text{SOR}}}}$ did not show any clear trend with different radionuclides (Table 8). The largest values of $\% {\text{STD}}_{{{\text{SOR}}}}$ were recorded on the Inveon system using ^89^Zr (106.38% in air, 240.08% in water). The RAYCAN system had the lowest values of $\% {\text{STD}}_{{{\text{SOR}}}}$ across all radionuclides (range 6.83–8.75% for water, range 7.64–10.42% for air).

## Discussion

We performed a NEMA image quality evaluation using a well-established measurement and analysis protocol and investigated the variations in image quality and quantification with four different radionuclides on single-system setting, by using three small-animal PET/CT systems of different vendors. We used the standard NEMA protocol with 20 min acquisition time for ^18^F and a radionuclide-specific acquisition time for the non-^18^F radionuclides. Radionuclide-specific, acquisition-specific and system-specific effects were shown to affect the PET image. To our knowledge, this was the first time that the effect of physical properties of different radionuclides on the image quality parameters were investigated using three preclinical PET/CT imaging systems, in a single-center setting.

### Analysis of the %RC

The behavior in %RC followed a similar trend with different radionuclides on different small animal PET/CT systems (Fig. 2). Long- (^68^Ga) and short-range (^18^F, ^11^C, ^89^Zr) positron emitters within all systems could be separated in agreement with previous results [9]. ^68^Ga showed the lowest %RC on all of the PET/CT systems (Fig. 2), indicating dependency on positron range. ^18^F had the highest recovery from rod sizes of 1 to 5 mm while ^11^C and ^89^Zr fall in between on Inveon and Molecubes system. This trend was similar for the RAYCAN system with rod sizes of 4 mm to 5 mm. The maximum %RC with different radionuclides varies across the systems for each rod (Fig. 2), which is expected as differences in %RC are also due to the chosen reconstruction algorithms and parameters, especially for the short-range positron emitters [9]. We also saw a dependency of %RC on acquisition time (Additional file 1: Data S1).

PSF reconstruction was applied only on the RAYCAN system, as this was the only iterative algorithm available. The other systems used non-PSF reconstruction. When using PSF reconstruction, higher %RC values are expected than with non-PSF reconstruction. However, for most rod sizes, RAYCAN has the lowest %RC (Fig. 2). This is explained by the following factors. First is the relatively low resolution of the RAYCAN system compared to other systems, positron range and to the size of the rods. Secondly, the %RC is calculated based on the mean of the line profile across the rod, reducing potential overshoot effects due to PSF. Thirdly, a low number of iterations (2) with a post-filter were used for PSF reconstruction, reducing potential overshoot effects, which are more prominent with a high number of iterations and without filtering.

Thus, the %RC seems to be dependent on system sensitivity and resolution in regard to the radionuclide positron fraction, physical half-life and positron range. Therefore, the individual differences in maximum %RC between the systems are explained not only by radionuclide-specific qualities but also by system-specific performance qualities, such as the sensitivity, intrinsic resolution and the implemented reconstruction algorithm. The %RC value is then affected by the following: (1) counting statistics, reflecting image noise and system sensitivity (2) resolution differences and the image reconstruction algorithm (3) positron range, physical half-life and positron fraction of the radionuclide which affect the two former factors. The dependency on %RC on the counting statistics is more prominent with systems with low sensitivity and radionuclides with low positron fractions and physical half-life compared to ^18^F.

### Analysis of the $\% {\text{STD}}_{{{\text{RC}}}}$

The $\% {\text{STD}}_{{{\text{RC}}}}$ reflected the radionuclide- and system-specific qualities as the %RC evaluation (Fig. 3). ^68^Ga showed the highest variability with the 1 mm rod size across all systems whereas ^18^F and ^89^Zr had the lowest variability on two systems. The systems included have different resolution characteristics, with 1.9 mm in RAYCAN and 1.8 mm in Inveon up to 1.1 mm in Molecubes [15–17]. The intrinsic resolution of the imaging system versus the positron range of the radionuclide will then be reflected in this parameter. Noise from the image acquisition in regard to positron fraction, physical half-life, used acquisition time, sensitivity and system hardware design will affect $\% {\text{STD}}_{{{\text{RC}}}}$, where increased noise will result in increase of $\% {\text{STD}}_{{{\text{RC}}}}$. A related parameter is then the phantom %SD measured from the uniform compartment, which means that the higher the noise, the higher the %SD and the higher the $\% {\text{STD}}_{{{\text{RC}}}}$ (Table 6, Fig. 3). Additional contribution is given by the reconstruction algorithm and its noise handling properties, although a lower $\% {\text{STD}}_{{{\text{RC}}}}$ can be achieved only if the total number of events is large enough to exclude the effect of contribution of Poisson processes.

In summary, the following factors contribute to the radionuclide-specific variability in the $\% {\text{STD}}_{{{\text{RC}}}}$ parameter. The factors include positron range, resolution of the system, positron fraction and physical half-life, acquisition time and sensitivity and noise originating from the reconstruction. Some variability is introduced by the measurement and analysis procedure, as parameter includes measurement of activity across the whole rod.

### Analysis of the %SD

We detected the smallest variation in %SD within each system with all values within range of 2% among different radionuclides (Table 6). Disselhorst et al. found that the largest differences in %SD originate from various reconstruction algorithms for the same radionuclide, when using radionuclide-specific acquisition times and a single PET/CT system [9]. Similarly to %RC, we also saw a dependency of %SD on the acquisition time, as expected (Additional file 1: Data S1). Thus, the %SD remains relatively stable between radionuclides as long as sufficient amount of counting statistics is collected and the same reconstruction algorithm is applied (Table 6). If the total activity or the counting statistics is too low, the standard deviation in the uniform region and other phantom regions will be affected by the relative sensitivity of the systems.

As the positron fraction, physical half-life differences and the system sensitivity with the reconstruction algorithm are reflected by this parameter, a large variation was seen between the systems (range 4.85–8.46%). We also noticed a bias in the Molecubes system, which shows the lowest mean activity for ^68^Ga, ^18^F and ^89^Zr. Given the %SD values are higher than for other systems, we suspect that this is not caused by difference in noise but a calibration offset. However, we were unable to verify this given we do not currently have access to the raw calibration factors on the system.

### Analysis of image quantification

We detected large differences in quantification between the systems and different radionuclides (Table 7). One system showed a systematic overestimation of the activity (Inveon), whereas the bias fluctuated between the other two systems in both positive and negative direction (RAYCAN and Molecubes).

The large variation of the quantification accuracy is attributed by several factors. The first is the need of a proper calibration protocol specifically for each radionuclide across the systems, to minimize the over- and underestimations between the systems. Two systems (RAYCAN and Inveon) also showed absolute fluctuations over 5% with ^18^F. One would expect that since the systems are calibrated with ^18^F, the absolute errors would be within the expected fluctuations of ± 5%. This deviation might be caused by the differences in the calibration procedures between the systems, error in the measurement of exact target dose in the calibrator and limited accuracy of the activity measurement in images from the small compartment of the phantom. Other potential factors would be a drift in the system since the time point of the calibration or a difference in the reconstruction, activity, or the geometry between calibration phantom and the NEMA phantom.

Given that the system-specific calibration procedures use different sizes of phantoms or syringes between the systems, which have different geometry and volume than the NEMA phantom and the activities used for calibration (5 MBq to 20 MBq) are different from the NEMA specified activity (3.7 MBq), fluctuations are expected. It would be beneficial to use a single phantom with the same geometry to derive calibration factors between the systems to minimize the variation. Finally, changes e.g. in reconstruction parameters can result in differences larger than the expected fluctuation of ± 8.4 kBq/mL. This expected fluctuation can be calculated using the NEMA-recommended initial activity (3.7 MBq), the volume of the phantom (22 mL) and the allowed fluctuation (± 5%) in activity. Together, this produces an expected activity concentration of 168 kBq/mL from which the allowed fluctuation in units of kBq/mL can be derived by using the allowed fluctuation in percentage units.

### Analysis of SOR

The behavior in SOR varied more between the systems (Table 8). Both ^11^C and ^68^Ga had SOR larger than ^18^F and ^89^Zr in water on all systems. Thus, differentiation of short- and long-range positron emitters similarly to [9] could be established in the water compartment across all systems. For SOR in air, ^11^C and ^68^Ga had larger SOR compared to than ^18^F and ^89^Zr on Molecubes and RAYCAN. On RAYCAN, only ^68^Ga could be differentiated clearly from other radionuclides based on SOR (SOR in air 0.25, SOR in water 0.32). ^18^F and ^89^Zr—the radionuclides with the lowest positron ranges—showed the lowest spill-over rations with all three systems.

Disselhorst et al. differentiated three factors which affect SOR: positron range, system-specific data corrections and the dimensions of the cold cylinder regions of the phantom. The authors suggested not to use SOR in water as data correction performance in assessment of radionuclides with long positron range (9.2 mm for ^68^Ga) as with these radionuclides the SOR in water is caused by positrons emitted from the main body of the phantom, which annihilate in the water-filled compartment. The same effect is seen in our measurements with both ^68^Ga and ^11^C which show higher SOR in water compared to other radionuclides (Table 8). Thus, the SOR in water reflects a mixture of the effects of the data corrections and the positron range, although variations between radionuclides and systems are evident.

There is a large variation between the radionuclides system-wise in the SOR in air, explained by the differences in system-specific implementation of corrections for randoms and scatter in the image reconstruction. As the air compartment can be used as an indicator the system-specific data correction performance [9], Inveon seems to be most effective in correcting the activity in the air compartment, showing lowest SOR in air across all radionuclides. The large SOR measured on RAYCAN system are due to missing data corrections for randoms and scatter, making this performance value not comparable against the two systems with data corrections implemented. These factors indicate, that for standardization purposes, the SOR in air would be the most challenging to match between the systems, as data correction implementations and their effects are very system-dependent.

### Analysis of $\% {\text{STD}}_{{{\text{SOR}}}}$

The last parameter which we quantified was $\% {\text{STD}}_{{{\text{SOR}}}}$. This parameter measures the variation in the cold compartments in the phantom versus the uniform compartment, and showed no radionuclide-specific trend (Table 8). Based on our results, this parameter is challenging to use as an indicator of the radionuclide-specific qualities and might be biased when the activity in the cold region is low. This is shown in our measurements using the Inveon system, which show the highest $\% {\text{STD}}_{{{\text{SOR}}}}$ of all of the measurements and very high values (106.38% and 240.08%) with ^89^Zr. However, based on the SOR value in air, the Inveon should have most efficient data corrections in place of all the systems evaluated.

The high $\% {\text{STD}}_{{{\text{SOR}}}}$ on the Inveon system is caused by measuring a very low mean value versus high standard deviation inside the cylinder VOI. The low mean is caused by effective data correction, as shown by the low SOR with this radionuclide, whereas small regions with high activity on otherwise cold regions increase the standard deviation. This effect results in high $\% {\text{STD}}_{{{\text{SOR}}}}$, as can be seen from Eq. (2).

Moreover, Eq. (2) shows that $\% {\text{STD}}_{{{\text{SOR}}}}$ increases in magnitude either with large standard deviation or low mean value in the cold compartment. In this case, a region with high SOR (high mean) but good uniformity (low standard deviation) would result in lower $\% {\text{STD}}_{{{\text{SOR}}}}$ than in the opposite case. This is evident with the RAYCAN system, which showed the lowest $\% {\text{STD}}_{{{\text{SOR}}}}$ for all radionuclides (Table 8), across all systems, although the data was not corrected for scatter or randoms.

Theoretically, an efficient data correction would result both in low mean value and low standard deviation in the cold region, resulting to effective negation of spill-over of activity and any residual activity inside the compartment. Whereas it seems that in our measurements with the Inveon system, a low mean and large standard deviation are occurring simultaneously, due to high and low activity regions, which increases $\% {\text{STD}}_{{{\text{SOR}}}}$. This explains the difference in $\% {\text{STD}}_{{{\text{SOR}}}}$ between Inveon and other systems. However, it is not guaranteed that both the standard deviation and the mean value in the compartments are connected, that is, they are always increasing or decreasing by a similar amount or to the same direction. Therefore, due to these factors affecting to $\% {\text{STD}}_{{{\text{SOR}}}}$ calculation, we recommend that this parameter should be always investigated in connection with the SOR whenever the effectiveness of data correction performance is evaluated, with radionuclides with positron ranges different from ^18^F.

### Comparison of results to previous studies

Previously, multi-radionuclide evaluations on preclinical PET/CT systems have been performed on the ALBIRA II, ARGUS, Mediso nanoScan, Inveon and Molecubes systems. We’ve collected the %RC and %SD results from these evaluations to Table 9, where available. Attarwala et al. compared ^18^F, ^68^Ga and ^64^Cu and reported lower %RC for ^68^Ga (< 60%) with similar %SD (~ 6%) as compared to ^18^F [11]. Cañadas et al. performed an evaluation using ^68^Ga and ^18^F using the ARGUS system, reporting %RC for ^68^Ga in range of 0.17 and 0.72 in comparison to 0.28 to 0.92 with ^18^F [12]. The reported %SD were high for both nuclei (> 15%), possibly due to applying a large number of image updates. Using increased acquisition time for both ^18^F and ^68^Ga, Gaitanis et al. reported %RC for ^68^Ga in range of 0.09 to 0.60 compared to 0.18 to 0.87 with ^18^F [13]. For the Inveon system, Disselhorst et al. reported %RC in the range of 0.1 to 0.6 with ^68^Ga, 0.2 to 1.0 with ^18^F and 0.2 to 0.9 with ^89^Zr, with lower %SD than our study (2% to 3%) [9]. The %RCs measured in [9] agree very well with our results with ^68^Ga, ^18^F and ^89^Zr for the Inveon system.

**Table 9:** Comparison of %RC and %SD values reported from previous multi-radionuclide studies

System	Radionuclide	Activity (MBq)	Acquisition time (min)	Algorithm	Iterations/updates	%RC 1 mm	%RC 2 mm	%RC 3 mm	%RC 4 mm	%RC 5 mm	%SD	References
ALBIRA II	^18^F	8	15	MLEM	25	N/A	0.31	0.60	0.76	0.79	6.4	[11]
	^68^Ga	8	15	MLEM	25	N/A	0.10	0.30	0.42	0.56	6.7	
ARGUS	^18^F	3.7	20	FORE-2D OSEM	48	0.28	0.8^a^	0.9^a^	1.0^a^	0.92	15.1	[12]
	^68^Ga	3.7	20.88	FORE-2D OSEM	48	0.17	0.3^a^	0.4^a^	0.5^a^	0.72	17.0	
Mediso nanoScan	^18^F	3.7	30	Tera-Tomo 3D	52	0.18	0.46	0.68	0.79	0.87	5.0^a^	[13]
	^68^Ga	4.19	30	Tera-Tomo 3D	52	0.09	0.20	0.36	0.48	0.60	5.0^a^	
Inveon	^18^F	3.7	20	3D-OSEM MAP	2/18	0.2^a^	0.4^a^	0.7^a^	0.8^a^	1.0^a^	2^a^	[9]
	^68^Ga	3.7	22.88	3D-OSEM MAP	2/18	0.1^a^	0.2^a^	0.4^a^	0.7^a^	0.6^a^	3^a^	
	^89^Zr	3.7	80.4	3D-OSEM MAP	2/18	0.2^a^	0.4^a^	0.6^a^	0.8^a^	0.9^a^	2^a^	
Molecules	^18^F	4–8	20	3D-OSEM	100	0.3	0.79	0.85	1.01	0.97	N/A	[14]
	^11^C	4–8	30	3D-OSEM	100	0.52	0.56	0.86	0.93	1.02	N/A	
	^68^Ga	4–8	20	3D-OSEM	100	0.11	0.35	0.46	0.56	0.57	N/A	

*MLEM* maximum-likelihood expectation–maximization,* FORE-2D OSEM* Fourier rebinning followed by 2D ordered-subsets expectation-maximization,* 3D-OSEM MAP* 3D ordered-subsets expectation-maximization in combination with maximum a posteriori,* 3D-OSEM* 3D ordered-subsets expectation-maximization

^a^Values have been derived from the reference Figures and are approximate, N/A = the value is not reported in the reference

Although there are differences in the system performance, the reconstruction algorithms, reconstruction parameters, acquisition times and activities between this study and previous investigations (Table 9), some comparisons can be performed. Of note are the %RC results for ^68^Ga with other systems (Table 9), where the %RCs are very comparable (maximum values 0.56 to 0.72) to our results. In terms of %SD, the ALBIRA II and Mediso NanoScan show comparable values (approximately 5% to 6.7%) to our results with both ^68^Ga and ^18^F. However, the %SD values measured from the ARGUS system and from the Inveon system differ from ours, due to the amount of iterations used for reconstruction on both systems. ARGUS uses a relatively high number of iterations (48), whereas in Inveon the amount of image updates is lower (2/18) than in our study (18/16).

In comparison of our results with Molecubes, Presotto et al. compared 3 radionuclides with the Molecubes system, including ^18^F, ^11^C and ^68^Ga, where higher %RC were reported for ^11^C and ^18^F similarly in our study, with lowest %RC for ^68^Ga (< 0.6) [14]. The difference of %RC to our results is explained by the amount of iterations used as seen from our Supplemental data with the Molecubes system (Additional file 1: Fig. S4), where %RC of 0.3 for ^18^F and 1 mm rod can be reached when using 100 iterations, as in Presotto et al. For the RAYCAN system, only one previous evaluation exists [17], performed with ^18^F only, and with agreeable values to what are achieved in this paper. Thus, the results presented in this paper agree well with the studies published with the previous systems, concerning ^68^Ga and ^18^F, although there are variations in reported %RC and %SD due to different acquisition times, reconstruction parameters and algorithms used in the measurements, prompting for a standardized approach for conducting the measurements, image reconstructions and evaluations.

### Limitations

There are limitations imposed by the phantom, using the NEMA protocol for quantification of different parameters with different radionuclides and in applying the phantom measurements to in-vivo data. A recent paper has discussed in detail about the challenges in the NEMA measurements [22], from which we will focus only on the part which concern the image quality measurements. First is the construction of the phantom, where rods used to quantify the %RC are embedded in a cold background, whereas %SD and SOR are quantified from regions with hot background. This means that the phantom does not perfectly mimic the in-vivo situations where hot targets are usually located in a region with background activity. This also affects the evaluation and comparison of %RC among different reconstruction algorithms in the phantom and in-vivo as reconstruction convergence is also dependent on the level of background activity [23].

The second limitation is imposed by the NEMA measurement protocol used to quantify the %RC of the rods. As Hallen et al. discussed, the %RC actually measures a combination of recovery and variance over the rods [22]. The calculation of %RC includes measurement of the maximum activity in a ROI, which causes a positive correlation of noise and recovery coefficients. The NEMA protocol specifically states to search for the maximum pixel value in each of the rods from an averaged image, and then draw line profiles over the rods from which the %RC is calculated [22]. This may introduce inaccuracies in the presence of low counting statistics or noise, which will in turn create a positive bias for the %RC measurement with high-noise, low-statistics data.

To study this positive bias in %RC, we repeated the acquisitions with ^11^C and ^68^Ga using a 20 min acquisition time (Additional file 1: Data S1). The effect of lower counting statistics can be seen well with ^11^C and ^68^Ga which show increased %RC when using 20 min acquisition time (Additional file 1: Fig. S3), indicating that %RC is positively biased due to lower counting statistics. When using radionuclide-specific acquisition times, the %RCs are lower (Additional file 1: Fig. S3, Table S1). The effect of producing considerably smoother images with low variance will also positively bias the %RC with the larger rod sizes, as seen from the Supplemental Data (Additional file 1: Tables S5 to S7).

In addition, the measurement of absolute quantification should be performed using a different type of phantom (e.g. a large uniform phantom) as the NEMA protocol does not specifically state any measurement of absolute quantification from the uniform compartment of the phantom. Using the uniform compartment as a measure of absolute recovery might be limited in accuracy, giving only a rough estimate of the quantitative accuracy of the systems. There are also additional measurements which could be considered e.g. acquiring the phantom with multiple off-axis positions to study the effect of resolution non-uniformity with multiple radionuclides. In these cases, the measurements would need to be repeated with PSF correction turned on and off as PSF correction tends to reduce the resolution non-uniformity across the FOV. However, the NEMA standard does not specifically recommend in performing off-axis image quality measurements.

Finally, the comparison between systems in this study is hampered due to different sensitivities and that specific calibration factors need to be applied between the systems to enable an unbiased comparison. This would enable a more straightforward comparison of system-to-system performance. Thus, the protocol used in this study allows to study the effect of radionuclides reliably only within a specific system. To account for the different sensitivities for the systems in the study, adjustment of the acquisition time experimentally according to the sensitivity of the systems might be needed. As can be noted from the acquired total counts collected afterward from each of the system, decreasing the acquisition time in Molecubes and increasing the acquisition time for RAYCAN by a factor might allow to compensate for the sensitivity differences between the systems. Another option would be for the manufacturer of each of the system to implement a protocol to acquire by counts on all of the systems. A specific calibration protocol should be applied beforehand to take into account not only the different sensitivities but also calibration differences, reconstruction and data corrections implemented in the systems.

Furthermore, as all of the systems are calibrated routinely using ^18^F only, a radionuclide-specific calibration protocol might be desired and the systems should be cross calibrated before assessment of quantification results between the systems. This is reflected by our quantification results (Table 7), where variation is high between systems even with ^18^F. However, in routine preclinical or clinical imaging the calibrations are generally performed with either ^18^F only or a ^68^Ge solid phantom and there are no specific calibration factors for each radionuclide, although they might be beneficial for quantitative accuracy.

### Future directions

In summary, the factors that greatly contribute to the PET image quality and the parameters estimated were the radionuclide-specific positron fraction, physical half-life and positron range. Variation is introduced by system-specific qualities, including system sensitivity, spatial resolution, reconstruction algorithm and the implemented data corrections for randoms and scatter. In our supplemental data (Additional file 1: Data S1), we’ve highlighted that %RC measurements with non-^18^F radionuclides will be biased unless the differences in positron fraction and physical half-life are accounted for. Moreover, in line with the studies of Disselhorst et al. and Liu et al., we determined that the positron range is a limiting factor concerning recovery of small targets (%RC) and the resulting spill-over in cold regions surrounded by activity [9, 10]. These effects were most evident with ^68^Ga.

The limitations imposed by positron range become more evident with the increasing resolution of modern preclinical PET/CT systems and with radionuclides with long positron range such as ^68^Ga. Therefore, a performance benefit would be gained from a method for positron range correction. Currently, none of the systems have such method available. So far, positron range corrections have been used in research settings, where methods are based on deburring with an appropriate kernel in the reconstruction. For short review on the methodologies, we refer to two recent papers [24, 25]. In short, methods using spatially-invariant kernels are simple to implement, but are mainly effective for uniform media [24]. For heterogeneous media, spatially-variant anisotropic kernels need to be implemented in addition to taking into account the different densities of tissues [25]. Up to this date, the authors are currently aware only of one study [26] which applied a positron range correction method on a preclinical PET/CT and in-vivo data.

The results presented indicate that there is still considerable room for both optimization and standardization when using different radionuclides. In terms of optimization, selected reconstruction parameters e.g. image matrix size, filtering and the amount of iterations will affect the %RC, %SD and SOR parameters (Additional file 1: Data S2). As for our evaluation, we applied the default parameters, although they varied between the systems. We believe that our results are to be more reflective of imaging performance at baseline, thus we did not tune these parameters to suit specifically for different radionuclides or systems. Further optimization studies to achieve an optimal image quality with different radionuclides are encouraged. In practice, tuning the different reconstruction options suitable among systems might be limited depending on the available options on the system. In general, few modifiable options are available, such as matrix or pixel size, algorithm, the amount of iterations and filtering.

Concerning standardization, we believe that the variation seen with the maximal %RC and the %SD in the uniform compartment could be greatly reduced if a specific harmonization protocol was applied. By harmonizing calibrations, acquisition, image reconstruction parameters and possibly by post-processing (e.g. filtering) of the images, both the noise structure, contrast recovery and resolution properties could be made more uniform among different imaging systems. For example, ensuring that sufficient amount of counting statistics are collected, reconstructing images with lower matrix size and applying filters would result in reduced %SD. However, as stated by Hallen et al., the optimal %RC would need to be determined carefully with comparison of uniformity, as the overall image quality performance is a trade-off between uniformity and recovery [22]. This can be seen from the Molecubes data reconstructed with different reconstruction options, two different algorithms and variable amount of iterations (Additional file 1: Fig. S4, Data S2). As this will require a careful study of the effect of different combination of reconstruction parameters to the NEMA image quality metrics on each system and each radionuclide specifically, further studies on the effect of harmonization on both the acquisition and reconstruction parameters are encouraged to minimize the variation between different preclinical systems in multi-radionuclide studies.

## Conclusions

Our study has highlighted several factors, which affect the image quality parameters when using the preclinical PET/CT systems for multi-radionuclide imaging and NEMA image quality measurements at baseline performance. These factors need to be addressed in further standardization attempts between different radionuclides, different PET/CT systems and when using the NEMA image quality protocol. System-, acquisition and radionuclide-dependent qualities were identified to affect the image quality parameters measured by the NEMA image quality protocol and should be accounted for by applying a specific calibration and harmonization protocol when conducting multi-center or multi-system studies in preclinical imaging. As this study was performed only as a single-center and single-system evaluation setting, further attempts for harmonizing system performance in multi-system and multi-center studies are needed.

In general, we have noted that most of the systems performed most optimal in terms of NEMA image quality parameters when using ^18^F, where ^11^C and ^89^Zr performed slightly worse than ^18^F and the performance was least optimal when using ^68^Ga. As ^68^Ga produced the lowest %RC, largest $\% {\text{STD}}_{{{\text{RC}}}}$ and increased SOR, further optimization of system performance would be beneficial for ^68^Ga as well as radionuclides with long positron ranges. As modern preclinical PET/CT systems are close to sub-millimeter resolution, it is important to take into account the positron range to improve image quality with these radionuclides. A large variation of image quantification were also seen between the systems, which would also prompt further optimization not only in terms of improving the image quality, but also by improving image quantification. This would require assessment of the accuracy of system calibration, data corrections and of image quantification with different radionuclides.

## Supplementary information

**Additional file 1: Fig. S1**. Construction scheme of the NEMA phantom with a photograph of the phantom. **Data S1**. Effect of using the standard 20 minute acquisition time for ^11^C and ^68^Ga. **Data S1, Table S1**. Recovery coefficients of the phantom rods with different radionuclides and acquisition times. **Data S1, Table S2**. Percentage standard deviation of recovery coefficients of the phantom rods with different radionuclides and acquisition times. **Data S1, Table S3**. The mean activity and the percentage standard deviation measured from the uniform compartment of the phantom with different acquisition times. **Data S1, Fig. S2**. Image quality comparison for ^11^C and ^68^Ga using the 20 minute acquisition time. **Data S1, Fig. S3**. Recovery coefficients with ^11^C and ^68^Ga only. **Data S2**. Effect of different reconstruction algorithms and parameters. **Data S2, Table S4**. Image reconstruction parameters and data corrections used for the reconstruction evaluation. **Data S2, Fig. S4**. Dependency of image quality parameters on the amount of iterations. **Data S2, Table S5**. Recovery coefficients of the phantom rods with different radionuclides and reconstruction options. **Data S2, Table S6**. Percentage standard deviation of recovery of the phantom rods with different radionuclides and reconstruction options. **Data S2, Table S7**. The mean radioactivity with percentage standard deviation and relative difference in the uniform compartment with the spill-over-ratios and their percentage standard deviations with different reconstruction schemes..

## Data Availability

The datasets used and analysed during the current study are available from the corresponding author on reasonable request.

## Abbreviations

%RC: Percent recovery coefficient
%STD, %SD: Percentage standard deviation
[^18^F]FDG: [^18^F]Fluorodeoxyglucose
3D: Three-dimensional
FOV: Field-of-view
FBP: Filtered backprojection
GPU: Graphics processing unit
LSO: Lutetium oxyorthosilicate
LYSO: Lutetium–yttrium oxyorthosilicate
NEMA: National electrical manufacturers association
OSEM: Ordered subsets expectation maximization algorithm
PET/CT: Positron emission tomography/computed tomography
PSF: Point spread function
SOR: Spill-over ratio
SP-MAP: Shifted poisson maximum a posteriori reconstruction
VOI: Volume of interest
