# Supplementary material for: Evaluation of image quality with four positron emitters and three preclinical PET/CT systems
Source: EJNMMI Res. 2020 Dec 10;10:155. doi: 10.1186/s13550-020-00724-z (PMC7728905; doi:10.1186/s13550-020-00724-z)
Supplement: Supplementary file 1 — Additional file 1: Fig. S1. Construction scheme of the NEMA phantom with a photograph of the phantom. Data S1. Effect of using the standard 20 minute acquisition time for 11C and 68Ga. Data S1, Table S1. Recovery coefficients of the phantom rods with different radionuclides and acquisition times. Data S1, Table S2. Percentage standard deviation of recovery coefficients of the phantom rods with different radionuclides and acquisition times. Data S1, Table S3. The mean activity and the percentage standard deviation measured from the uniform compartment of the phantom with different acquisition times. Data S1, Fig. S2. Image quality comparison for 11C and 68Ga using the 20 minute acquisition time. Data S1, Fig. S3. Recovery coefficients with 11C and 68Ga only. Data S2. Effect of different reconstruction algorithms and parameters. Data S2, Table S4. Image reconstruction parameters and data corrections used for the reconstruction evaluation. Data S2, Fig. S4. Dependency of image quality parameters on the amount of iterations. Data S2, Table S5. Recovery coefficients of the phantom rods with different radionuclides and reconstruction options. Data S2, Table S6. Percentage standard deviation of recovery of the phantom rods with different radionuclides and reconstruction options. Data S2, Table S7. The mean radioactivity with percentage standard deviation and relative difference in the uniform compartment with the spill-over-ratios and their percentage standard deviations with different reconstruction schemes.. [file 13550_2020_724_MOESM1_ESM.docx]

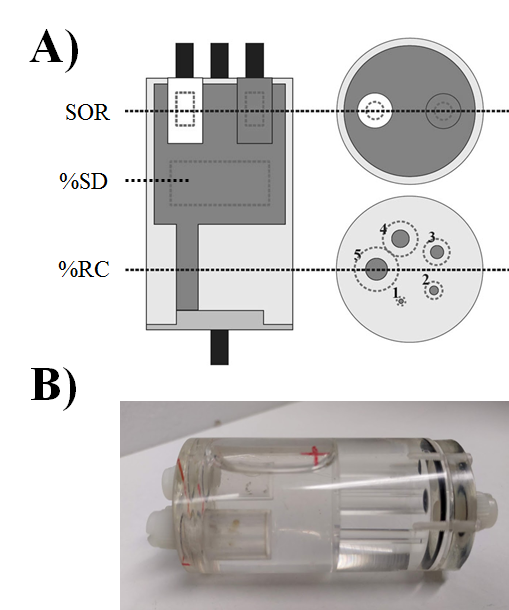


**Supplementary Figure 1. A)** Construction scheme of the NEMA phantom and regions where image quality parameters are quantified with **B)** a photograph of the phantom used in this study. Note that the photograph does not contain an actual measurement setup, as air is present. The construction scheme of the phantom was originally published in: Pajak MZ et al. *NEMA NU4-2008 Performance Evaluation of Albira: A Two-Ring Small-Animal PET System Using Continuous LYSO Crystals.* Open Medicine Journal. 2016;27;3. Available from: <https://openmedicinejournal.com/VOLUME/3/PAGE/12/>, licensed under CC BY-NC 4.0.

**Supplementary Data 1: Effect of Using the Standard 20 Minute Acquisition Time for ^11^C and ^68^Ga**

To investigate the effect of using the standard acquisition time in the NEMA protocol for non-^18^F nuclides, we repeated the study using an acquisition time of 20 minutes for ^11^C and ^68^Ga. The injected dose for ^11^C was 3.74, 3.66 and 3.73 MBq for RAYCAN, Inveon and Molecubes. For ^68^Ga, the dose was 3.32, 3.13 and 3.68 MBq. Image reconstructions and data analysis were performed similarly as with the nuclide-specific acquisition time. The numerical results are given in Supplementary Tables 1 to 3, in comparison to the nuclide-specific acquisition time. A visual comparison of the acquisition protocols with ^11^C and ^68^Ga is given in Supplementary Figure 2. A comparison of $\%RC$ with ^11^C and ^68^Ga using different acquisition protocols is shown in Supplementary Figure 3.

The results show higher $\%RC$ with the 20 minute acquisition time compared to nuclide-specific acquisition times for almost all rods (Supplementary Table 1). The highest $\%RC$ were measured on Raycan system with ^11^C with the exception of the 1 mm rod size (range 0.67 to 0.85) when using the 20 minute acquisition time. The mean increase of $\%RC$ with the 20 minute acquisition time across all rod sizes was 13.7 % and 8.0 % on Inveon, 1.45 % and 40.0 % on RAYCAN and 0.87 % and -2.72 % on the Molecubes system using ^68^Ga and ^11^C, respectively. This effect seems to be dependent on the system sensitivity and resolution, where the $\%RC$ differences are reduced with the largest rod sizes and with a system with highest sensitivity. On the Molecubes system, the effect of using a shorter acquisition time is not as clearly seen, attributed by the highest sensitivity (12.4 %) and resolution (1.1. mm) of the system. Using 20 minute acquisition resulted also in higher ${\%STD}_{RC}$across all systems (Supplementary Table 2), with the exception of ^11^C on the Molecubes system.

For the image uniformity evaluation, $\%SD$ increased when using the 20 minute acquisition time with ^68^Ga (mean increase 2.18 %) and ^11^C (mean increase 2.72 %), except for the Molecubes system with ^11^C and RAYCAN with ^68^Ga (Supplementary Table 3). ^68^Ga had the highest $\%SD$ on both the Inveon and Molecubes system. $\%SD$ values larger than 9 % were measured with ^11^C and the RAYCAN system when using 20 minute acquisition time.

These results indicate that the $\%RC$ is dependent on the acquisition time (Supplementary Figure 3) and reflects the sensitivity of the system. System-specific sensitivity differences were evident when using the 20 minute acquisition time with ^11^C and ^68^Ga, especially on the RAYCAN system (Supplementary Figure 3), where the $\%RC$ was positively biased. This effect was less evident with the Molecubes system (Supplementary Figure 3). This variation is attributed by the low sensitivity (1.7%) and the lowest resolution of the RAYCAN system (1.9 mm) compared e.g. to Molecubes. Using nuclide-specific acquisition times, ^11^C measurement on RAYCAN resulted in similar $\%RC$ as seen in other systems.

Increased noise in the images increased the values of ${\%STD}_{RC}$ (Supplementary Table 2) and $\%SD$ parameters (Supplementary Table 3) and $\%RC$, especially with the smallest rod sizes from 1 mm to 2 mm. The 20 minute acquisitions with ^68^Ga and ^11^C resulted in increased $\%SD$, except for ^68^Ga in RAYCAN and ^11^C in Molecubes.

In summary, using the standard 20 minute acquisition time for ^11^C and ^68^Ga resulted in higher $\%RC$ and $\%SD$, indicating parameter dependency on counting statistics. Variation in $\%RC$ and somewhat for $\%SD$ for nuclides other than ^18^F come also from the different positron fractions and half-lives. These have implications for performing NEMA image quality measurements with non-^18^F nuclides using the NEMA recommended 20 minute acquisition time, especially with low-sensitivity systems. $\%RC$ measurements with non-^18^F nuclides will be biased unless the differences in positron fraction and half-life are accounted for. Thus, nuclide-specific half-lives and positron fractions need to be accounted by increasing the image acquisition time to achieve comparable results in phantom measurements.

In conclusion, we’ve highlighted the importance of using nuclide-specific acquisition times for unbiased comparison of $\%RC$ and $\%SD$ between different nuclides in this supplementary data.

**Supplementary Table 1.** Recovery coefficients ($\%RC$) of the phantom rods with different nuclides.

| **System** | **Nuclide** | **Acquisition time** | **1 mm** | **2 mm** | **3 mm** | **4 mm** | **5 mm** |
| --- | --- | --- | --- | --- | --- | --- | --- |
| **RAYCAN** | 11C | 20 min | 0.13 | 0.67 | 0.91 | 0.87 | 0.85 |
|  |  | 32 min | 0.10 | 0.39 | 0.57 | 0.70 | 0.76 |
|  |  |  |  |  |  |  |  |
|  | 68Ga | 20 min | 0.06 | 0.14 | 0.29 | 0.42 | 0.61 |
|  |  | 26 min | 0.06 | 0.15 | 0.29 | 0.45 | 0.56 |
|  |  |  |  |  |  |  |  |
|  | 18F | 20 min | 0.07 | 0.34 | 0.61 | 0.76 | 0.85 |
|  |  |  |  |  |  |  |  |
|  | 89Zr | 72 min | 0.08 | 0.30 | 0.65 | 0.69 | 0.79 |
|  |  |  |  |  |  |  |  |
| **Inveon** | 11C | 20 min | 0.16 | 0.48 | 0.81 | 0.86 | 0.86 |
|  |  | 32 min | 0.14 | 0.49 | 0.69 | 0.83 | 0.83 |
|  |  |  |  |  |  |  |  |
|  | 68Ga | 20 min | 0.11 | 0.23 | 0.38 | 0.53 | 0.65 |
|  |  | 26 min | 0.07 | 0.23 | 0.38 | 0.52 | 0.60 |
|  |  |  |  |  |  |  |  |
|  | 18F | 20 min | 0.16 | 0.63 | 0.91 | 0.97 | 0.92 |
|  |  |  |  |  |  |  |  |
|  | 89Zr | 72 min | 0.13 | 0.53 | 0.76 | 0.86 | 0.88 |
|  |  |  |  |  |  |  |  |
| **Molecubes** | 11C | 20 min | 0.14 | 0.50 | 0.66 | 0.80 | 0.91 |
|  |  | 32 min | 0.16 | 0.50 | 0.71 | 0.78 | 0.88 |
|  |  |  |  |  |  |  |  |
|  | 68Ga | 20 min | 0.06 | 0.21 | 0.36 | 0.49 | 0.63 |
|  |  | 26 min | 0.07 | 0.21 | 0.34 | 0.50 | 0.62 |
|  |  |  |  |  |  |  |  |
|  | 18F | 20 min | 0.18 | 0.61 | 0.79 | 0.87 | 0.93 |
|  |  |  |  |  |  |  |  |
|  | 89Zr | 72 min | 0.11 | 0.48 | 0.71 | 0.82 | 0.87 |

**Supplementary Table 2.** Percentage standard deviation of recovery coefficients (${\%STD}_{RC}$) of the phantom rods with different nuclides.

| **System** | **Nuclide** | **Acquisition time** | **1 mm** | **2 mm** | **3 mm** | **4 mm** | **5 mm** |
| --- | --- | --- | --- | --- | --- | --- | --- |
| **RAYCAN** | 11C | 20 min | 0.24 | 0.15 | 0.14 | 0.11 | 0.10 |
|  |  | 32 min | 0.23 | 0.12 | 0.13 | 0.08 | 0.10 |
|  |  |  |  |  |  |  |  |
|  | 68Ga | 20 min | 0.73 | 0.15 | 0.27 | 0.19 | 0.16 |
|  |  | 26 min | 0.43 | 0.15 | 0.31 | 0.29 | 0.15 |
|  |  |  |  |  |  |  |  |
|  | 18F | 20 min | 0.22 | 0.19 | 0.07 | 0.13 | 0.07 |
|  |  |  |  |  |  |  |  |
|  | 89Zr | 72 min | 0.26 | 0.17 | 0.17 | 0.08 | 0.09 |
|  |  |  |  |  |  |  |  |
| **Inveon** | 11C | 20 min | 0.18 | 0.09 | 0.09 | 0.09 | 0.10 |
|  |  | 32 min | 0.12 | 0.09 | 0.08 | 0.09 | 0.09 |
|  |  |  |  |  |  |  |  |
|  | 68Ga | 20 min | 0.39 | 0.17 | 0.12 | 0.10 | 0.12 |
|  |  | 26 min | 0.37 | 0.17 | 0.10 | 0.11 | 0.08 |
|  |  |  |  |  |  |  |  |
|  | 18F | 20 min | 0.14 | 0.06 | 0.06 | 0.06 | 0.05 |
|  |  |  |  |  |  |  |  |
|  | 89Zr | 72 min | 0.17 | 0.07 | 0.07 | 0.07 | 0.07 |
|  |  |  |  |  |  |  |  |
| **Molecubes** | 11C | 20 min | 0.16 | 0.10 | 0.10 | 0.11 | 0.09 |
|  |  | 32 min | 0.23 | 0.14 | 0.13 | 0.13 | 0.14 |
|  |  |  |  |  |  |  |  |
|  | 68Ga | 20 min | 0.28 | 0.21 | 0.22 | 0.15 | 0.14 |
|  |  | 26 min | 0.36 | 0.12 | 0.12 | 0.13 | 0.11 |
|  |  |  |  |  |  |  |  |
|  | 18F | 20 min | 0.21 | 0.15 | 0.11 | 0.10 | 0.11 |
|  |  |  |  |  |  |  |  |
|  | 89Zr | 72 min | 0.18 | 0.11 | 0.11 | 0.11 | 0.11 |

**Supplementary Table 3.** The mean activity (kBq/mL) and the percentage standard deviation ($\%SD$) measured from the uniform compartment of the phantom.

| PET/CT  system | ^11^C  Mean activity (kBq/mL) | ^11^C  %SD | ^11^C  20 min  Mean activity (kBq/mL) | ^11^C  20 min  %SD | ^68^Ga  Mean activity (kBq/mL) | ^68^Ga  %SD | ^68^Ga  20 min  Mean activity (kBq/mL) | ^68^Ga  20 min  %SD |
| --- | --- | --- | --- | --- | --- | --- | --- | --- |
| RAYCAN | 190.13 | 5.81 | 143.94 | 9.31 | 168.90 | 6.91 | 142.67 | 6.42 |
| Inveon | 171.57 | 5.99 | 176.99 | 7.92 | 170.16 | 7.05 | 145.03 | 9.19 |
| Molecubes | 186.67 | 8.46 | 144.36 | 7.27 | 143.94 | 7.16 | 138.76 | 9.37 |


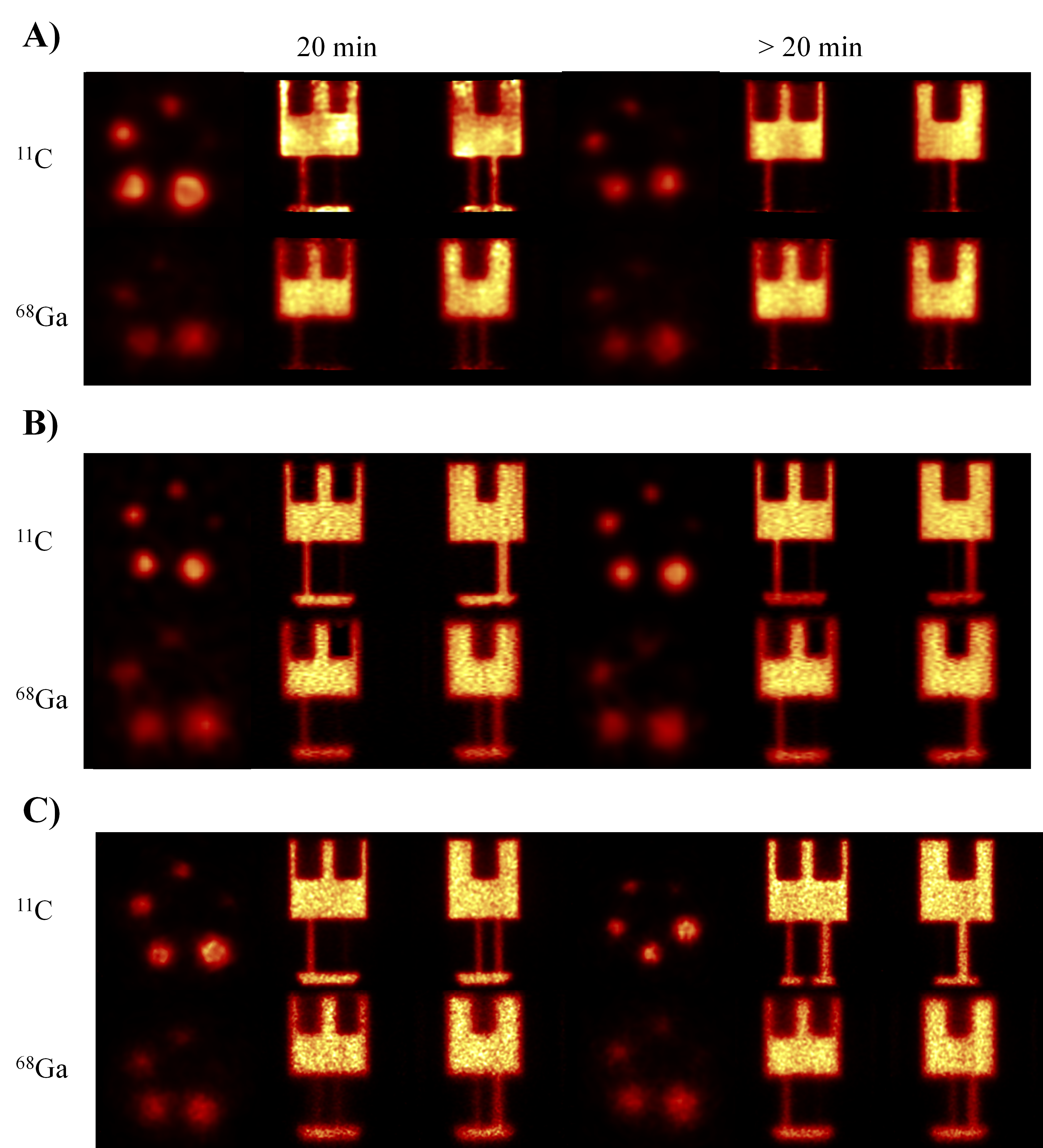


**Supplementary Figure 2.** Image quality comparison with **A)** RAYCAN **B)** Inveon and **C)** Molecubes with ^11^C and ^68^Ga using the 20 minute acquisition time specified by NEMA protocol and the nuclide-specific acquisition time (32 minutes for ^11^C and 26 minutes for ^68^Ga). Each image is scaled to 0 to 1.25 times the mean activity in the uniform compartment. A slight increase in image noise is seen with the 20 minute data.


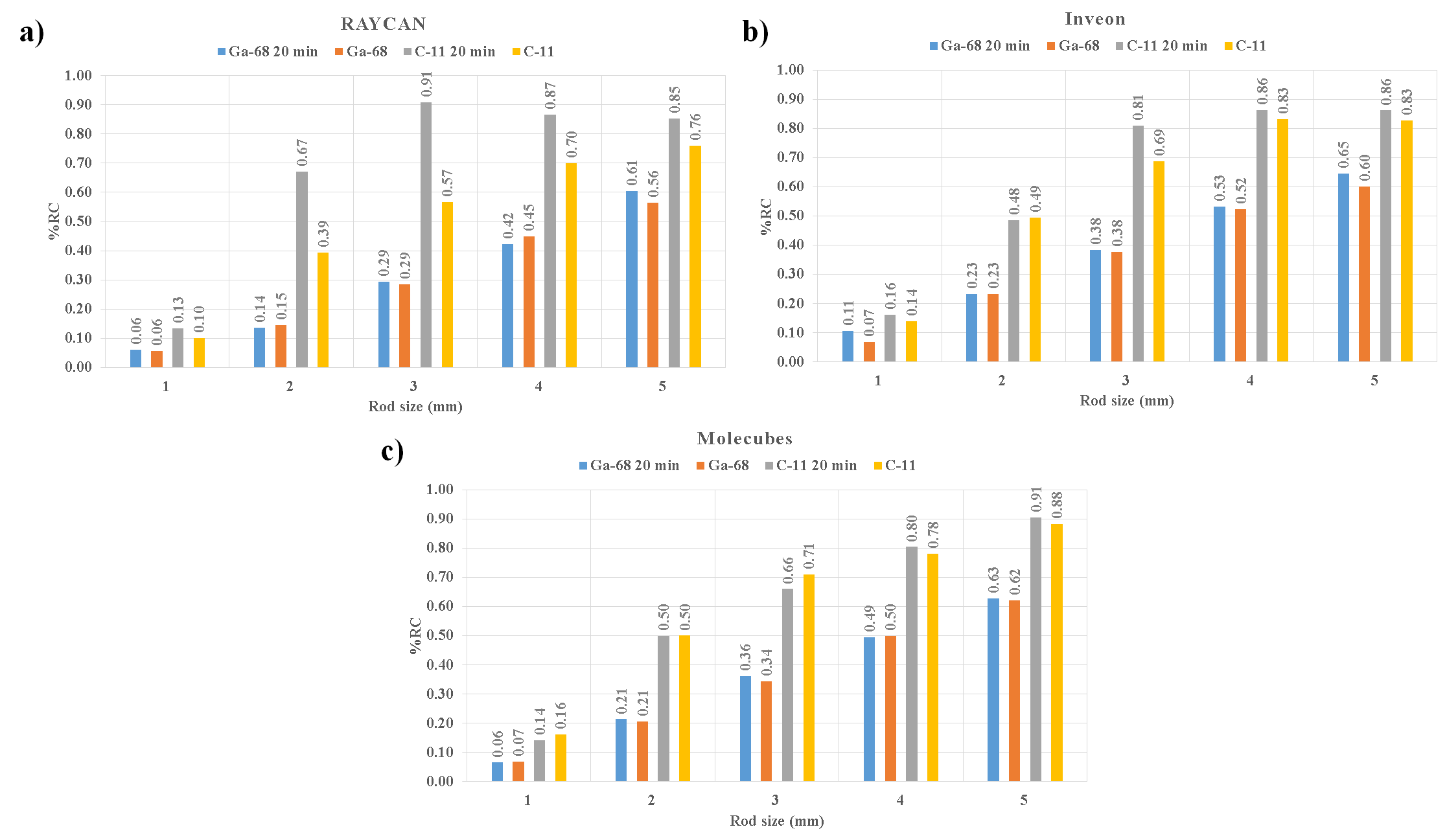


**Supplementary Figure 3.** Recovery coefficients ($\%RC$) with ^11^C and ^68^Ga only with the **a** RAYCAN, **b** Inveon and **c** Molecubes systems using both the NEMA specified acquisition time of 20 minutes and the nuclide-specific acquisition times. The effect of using nuclide-specific time is most evident with RAYCAN (lowest sensitivity) and less evident with Molecubes (highest sensitivity).

**Supplementary Data 2: Effect of Different Reconstruction Algorithms and Parameters**

To investigate the effect of applying different reconstruction algorithms and parameters using a single system, the data with nuclide-specific acquisition time from Molecubes was reconstructed using the standard GPU-based 3D ordered-subset expectation maximization reconstruction (*3D-OSEM Standard)* and a regularized version of the same algorithm (*3D-OSEM Regularized)* using a Huber prior [1]. The data was also reconstructed with smaller matrix size and smaller number of iterations (*3D-OSEM Smooth*) to reduce image noise. The parameters are summarized in Supplementary Table 4. The results of the evaluation with different parameters are shown in Supplementary Table 5 to 7.

Finally, the data was reconstructed using 10 to 100 iterations and standard parameters (*3D-OSEM Standard)*. The data with a different amount of iterations are shown in Supplementary Figure 4.

**References**

Hsu CH. An investigation of block-sequential algorithms in statistical PET image reconstruction. J Med Biol Eng 2004;24:77–83.

**Supplementary Table 4.** Image reconstruction parameters and data corrections used for the reconstruction evaluation. All data corrections were applied. No point spread function correction was applied.

| **Reconstruction algorithm** | **Matrix size** | **Pixel size** | **Iterations/ Subsets** | **Post-filter** | **β-value/**  **prior penalty** |
| --- | --- | --- | --- | --- | --- |
| 3D-OSEM  Standard | 192x192x384 | 0.4x0.4x0.4 | 30/7 | - | - |
| 3D-OSEM  Smooth | 92x92x192 | 0.8x0.8x0.8 | 20/7 | - | - |
| 3D-OSEM  Regularized | 192x192x384 | 0.4x0.4x0.4 | 30 | - | 0.2 |


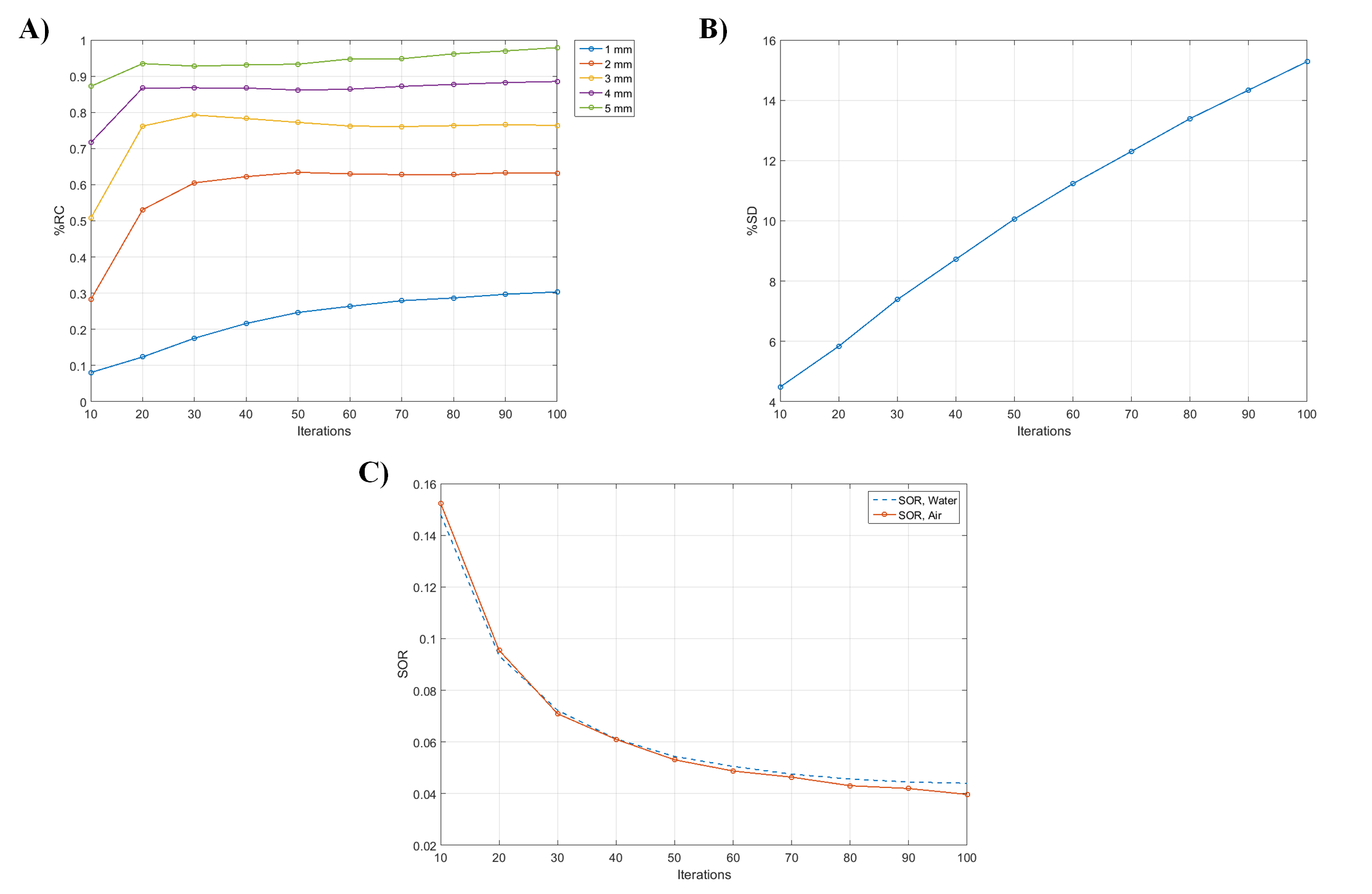


**Supplementary Figure 4. A)** Recovery coefficients ($\%RC$), **B)** percentage standard deviation in the uniform compartment $(\%SD)$ and **C)** spill-over ratios ($SOR$) with different amount of iterations (10 to 100 at 10 iteration intervals) using the Molecubes system and standard set of parameters (*3D-OSEM Standard*). The three parameters show a dependency on the amount of iterations, warranting for optimization studies in defining optimal parameters in terms of resolution, noise and contrast. Note also the inter-dependency of $\%RC$ and $\%SD$.

**Supplementary Table 5.** Recovery coefficients ($\%RC$) of the phantom rods with different nuclides and reconstruction options on the Molecubes system.

| **Algorithm** | **Nuclide** | **Acquisition Time** | **1 mm** | **2 mm** | **3 mm** | **4 mm** | **5 mm** |
| --- | --- | --- | --- | --- | --- | --- | --- |
| **3D-OSEM**  **Standard** | Ga-68 | 26 min | 0.07 | 0.21 | 0.34 | 0.50 | 0.62 |
|  | C-11 | 32 min | 0.16 | 0.50 | 0.71 | 0.78 | 0.88 |
|  | F-18 | 20 min | 0.18 | 0.61 | 0.79 | 0.87 | 0.93 |
|  | Zr-89 | 72 min | 0.11 | 0.48 | 0.71 | 0.82 | 0.87 |
| **3D-OSEM**  **Smooth** | Ga-68 | 26 min | 0.05 | 0.15 | 0.32 | 0.50 | 0.64 |
|  | C-11 | 32 min | 0.08 | 0.38 | 0.68 | 0.90 | 1.03 |
|  | F-18 | 20 min | 0.09 | 0.43 | 0.86 | 1.08 | 1.07 |
|  | Zr-89 | 72 min | 0.07 | 0.36 | 0.75 | 0.91 | 0.99 |
| **3D-OSEM**  **Regularized** | Ga-68 | 26 min | 0.05 | 0.18 | 0.32 | 0.47 | 0.62 |
|  | C-11 | 32 min | 0.11 | 0.41 | 0.63 | 0.77 | 0.90 |
|  | F-18 | 20 min | 0.11 | 0.48 | 0.71 | 0.86 | 0.93 |
|  | Zr-89 | 72 min | 0.08 | 0.41 | 0.64 | 0.79 | 0.85 |

**Supplementary Table 6** Percentage standard deviation of recovery coefficients (${\%STD}_{RC}$) of the phantom rods with different nuclides and reconstruction options on the Molecubes system.

| **Algorithm** | **Nuclide** | **Acquisition Time** | **1 mm** | **2 mm** | **3 mm** | **4 mm** | **5 mm** |
| --- | --- | --- | --- | --- | --- | --- | --- |
| **3D-OSEM**  **Standard** | Ga-68 | 26 min | 0.36 | 0.12 | 0.12 | 0.13 | 0.11 |
|  | C-11 | 32 min | 0.23 | 0.14 | 0.13 | 0.13 | 0.14 |
|  | F-18 | 20 min | 0.21 | 0.15 | 0.11 | 0.10 | 0.11 |
|  | Zr-89 | 72 min | 0.18 | 0.11 | 0.11 | 0.11 | 0.11 |
| **3D-OSEM**  **Smooth** | Ga-68 | 26 min | 0.13 | 0.09 | 0.10 | 0.07 | 0.06 |
|  | C-11 | 32 min | 0.07 | 0.14 | 0.09 | 0.08 | 0.05 |
|  | F-18 | 20 min | 0.12 | 0.10 | 0.07 | 0.07 | 0.06 |
|  | Zr-89 | 72 min | 0.12 | 0.11 | 0.08 | 0.08 | 0.06 |
| **3D-OSEM**  **Regularized** | Ga-68 | 26 min | 0.21 | 0.09 | 0.11 | 0.08 | 0.08 |
|  | C-11 | 32 min | 0.12 | 0.08 | 0.07 | 0.07 | 0.07 |
|  | F-18 | 20 min | 0.24 | 0.16 | 0.08 | 0.09 | 0.08 |
|  | Zr-89 | 72 min | 0.18 | 0.10 | 0.07 | 0.10 | 0.06 |

**Supplementary Table 7.** The mean activity with percentage standard deviation ($\%SD$) and relative difference (%Δ) in the uniform compartment with the spill-over-ratios ($SOR$) and their percentage standard devaitions ($\%STD$) with different reconstruction schemes.

| **Algorithm** | **Nuclide** | **Acquisition Time** | **Mean Activity** | **%SD** | **%Δ** | **SOR water** | **SOR air** | **%STD water** | **%STD air** |
| --- | --- | --- | --- | --- | --- | --- | --- | --- | --- |
| **3D-OSEM**  **Standard** | Ga-68 | 26 min | 143.94 | 7.16 | -14.87 | 0.12 | 0.09 | 13.78 | 13.78 |
|  | C-11 | 32 min | 186.67 | 8.46 | 11.29 | 0.13 | 0.17 | 13.04 | 19.50 |
|  | F-18 | 20 min | 174.46 | 7.39 | 3.73 | 0.07 | 0.07 | 15.85 | 12.14 |
|  | Zr-89 | 72 min | 154.20 | 7.34 | -7.82 | 0.07 | 0.06 | 16.71 | 13.61 |
| **3D-OSEM**  **Smooth** | Ga-68 | 26 min | 128.38 | 4.44 | -24.08 | 0.15 | 0.15 | 9.66 | 8.29 |
|  | C-11 | 32 min | 147.51 | 4.69 | -12.05 | 0.11 | 0.10 | 10.75 | 11.67 |
|  | F-18 | 20 min | 140.56 | 5.41 | -16.43 | 0.09 | 0.12 | 11.59 | 8.01 |
|  | Zr-89 | 72 min | 144.80 | 4.98 | -13.43 | 0.09 | 0.09 | 10.92 | 9.76 |
| **3D-OSEM**  **Regularized** | Ga-68 | 26 min | 141.38 | 4.79 | -16.39 | 0.11 | 0.10 | 10.67 | 9.75 |
|  | C-11 | 32 min | 129.65 | 4.68 | -22.70 | 0.09 | 0.06 | 10.00 | 15.90 |
|  | F-18 | 20 min | 173.48 | 5.37 | 3.15 | 0.07 | 0.07 | 13.09 | 9.83 |
|  | Zr-89 | 72 min | 190.51 | 4.86 | 13.89 | 0.07 | 0.07 | 10.19 | 10.30 |
